# Threat Effects and Work Incentives: Evidence From a Danish Sick Leave Reform

**DOI:** 10.1002/hec.70126

**Published:** 2026-07-08

**Authors:** Jeppe Elholm Madsen

**Affiliations:** ^1^ Department of Technology Management and Economics Technical University of Denmark Lyngby Denmark

**Keywords:** activation program, policy evaluation, regression discontinuity, sick leave reform, social insurance

## Abstract

This paper investigates the effects of a 2014 policy reform on paid sick leave in Denmark. The reform shortened the eligibility period from 56 to 26 weeks for paid sick leave and introduced a mandatory return‐to‐work activation program for individuals whose benefits were not extended. The reform initially led to a temporary increase in returns to employment, as individuals on sick leave resumed work to avoid entering the mandatory activation program. However, this effect fades in the long run, and no sustained employment improvements were observed.

## 1 Introduction

Paid sick leave provides financial support to workers who are temporarily unable to work due to illness or injury. The costs associated with sick leave benefits are considerable. For instance, in 2019, Denmark spent 1.6 billion EUR on paid sick leave. In the same year, the expenditure for unemployment insurance was 2.1 billion EUR, Statistics Denmark (2022). Similar program costs are observed in many Western countries. As populations age and sick leave claims increase, governments continue to reassess the generosity and structure of these programs to contain costs. Despite the financial importance of sick leave benefits, much less research has focused on their labor market effects compared to unemployment benefits. While extensive literature examines how unemployment insurance influences job search behavior, far less is known about how sick leave policies shape absence duration and return‐to‐work patterns.

When designing these programs, policymakers face a difficult trade‐off between supporting individuals during illness and encouraging timely returns to work. Longer benefit durations provide important security but may create moral hazard, as individuals could extend their absence beyond what is medically necessary. In contrast, shorter benefit durations reduce the risk of moral hazard by increasing the incentive to return to work once recovery allows, but they may also pressure some individuals to resume work before they are fully recovered.

This paper examines how mandatory activation programs—triggered by earlier reassessment of sick leave eligibility—affect the return‐to‐work rate of individuals on paid sick leave.

In particular, this paper addresses the following research question: How does earlier reassessment and mandatory activation affect the return‐to‐work rate of individuals on paid sick leave?

This paper contributes by (1) showing that sick‐listed individuals react to policy changes through a threat effect and (2) demonstrating that activation programs in themselves do not improve employment outcomes.

To provide evidence on the topic, I analyze a policy reform on paid sick leave in Denmark from 2014. The reform is prototypical in the sense that it carries many of the same features as reforms implemented in multiple OECD countries, making it a suitable case study for the purpose of the analysis. For example, Sweden's 2008 rehabilitation chain introduced fixed reassessments at 90, 180, and 365 days, with claimants transferred to activation if deemed able to work Regeringen (2007). In the same year, the United Kingdom replaced Incapacity Benefit with Employment and Support Allowance, requiring an assessment after 13 weeks to determine participation in work‐related activities Department for Work and Pensions (2008). Similarly, the Danish reform introduced a reassessment at 26 weeks, after which non‐extended cases entered mandatory activation, replacing the previous 56‐week rule. Individuals could enter paid sick leave either from employment or from unemployment. For those coming from employment, the reform primarily introduced earlier reassessment and mandatory activation, whereas for those entering from unemployment, it also implied a reduction in benefit generosity, as the activation payment was lower than the previous sick leave benefit.

This study provides evidence that earlier reassessment of sick leave eligibility and the introduction of a mandatory activation program increase return‐to‐work rates by up to 21% in the short run among individuals still formally employed. However, in the long run, the effects on employment appear to weaken, and there is no significant lasting impact. Interestingly, this is not due to the activation program itself, which fails to influence employment outcomes. Instead, the short‐run effect is primarily driven by behavioral responses around the time of the program enrollment. I interpret this as a “threat effect,” where individuals return to work to avoid being subjected to the activation program. This effect arises when individuals are informed that their sick leave period is ending and that they will soon be enrolled in the activation program. In essence, the policy introduces a cost of remaining on sick leave, making continued absence less attractive compared to returning to work.

Importantly, this effect is not driven by changes in benefit generosity, as the response is concentrated among those entering from employment—who were unaffected by the benefit reduction component of the reform—while individuals entering from unemployment show little, if any, short‐run effect and negative effects in the longer run. This supports the interpretation that the short‐run increase in employment reflects a behavioral response to the threat of activation rather than to reduced benefit levels.

The ability to identify these findings comes from access to detailed register data, which allows for weekly tracking of individual transitions from sick leave benefits to employment. This level of detail makes it possible to observe how policy changes influence return‐to‐work patterns with high precision. Each week, I can determine which benefits individuals receive or whether they have re‐entered employment, enabling a clear analysis of behavioral responses to policy shifts. Crucially, the data allows me to pinpoint the exact timing of the announcement informing individuals that their sick leave period will not be extended, allowing me to measure immediate behavioral responses to this information. Additionally, the Danish reform's structure allows me to apply a regression discontinuity design, leveraging the cut‐off around the policy implementation. This enables a comparison between two policy regimes: one where individuals were eligible for 56 weeks of paid sick leave, and another where eligibility was reduced to 26 weeks, with a mandatory return‐to‐employment program introduced after this period. This framework provides a strong basis for estimating the causal effects of activation programs on employment outcomes.

This study relates most to Rehwald et al. (2018), Ziebarth (2013), and Graversen and van Ours (2008) but extends their findings in important ways. Like Rehwald et al. (2018), I find that activation programs have little impact on long‐term employment outcomes for sick‐listed individuals. However, I show that these programs significantly increase public costs without improving employment. Consistent with Ziebarth (2013), my results indicate that sick‐listed workers are not highly responsive to financial incentives in the long run. Additionally, similar to Graversen and van Ours (2008), I document a threat effect—but whereas their study focuses on unemployment insurance, I show that sick‐listed individuals exhibit the same behavioral response. This effect arises when individuals realize their sick leave cannot be extended, prompting them to return to work to avoid activation.

The key contribution of this study is to identify the threat effect as the main driver behind an increased return to work among sick‐listed employees. While mandatory activation increases employment rates in the short run, this effect does not stem from improved job readiness or the effectiveness of the program itself. Instead, individuals return to work to avoid mandatory activation, mirroring responses seen in unemployment insurance. This suggests that returning to work while still unwell may be preferable to facing activation requirements.

A large body of research examines financial incentives and activation policies in unemployment insurance. Reducing benefit generosity shortens unemployment duration Lalive et al. (2006), while extending benefits improves job matches Nekoei and Weber (2017). Activation policies also play a role: Graversen and van Ours (2008) find that mandatory activation increases employment primarily through a threat effect, where individuals return to work to avoid participation rather than benefiting directly from activation programs. More broadly, Card et al. (2010) confirm that job search assistance increases short‐term employment, while long‐term training programs have more gradual effects.

For sick‐listed workers, research suggests that higher benefits increase absenteeism, while financial constraints reduce sick leave durations. Henrekson and Persson (2004) and Ziebarth and Karlsson (2010) find that higher sick pay generosity leads to greater absenteeism, while reducing employer compensation lowers absence rates. However, long‐term sick workers are less responsive to financial incentives: Ziebarth (2013) finds that cuts in sick pay had no significant impact on long‐term absenteeism, suggesting that moral hazard is more relevant for short‐term absences. Similarly, Dale‐Olsen (2014) finds that workers with private supplementary sick pay take more leave than those relying solely on public benefits, reinforcing heterogeneous behavioral responses to sick pay reforms.

Studies on activation programs provide mixed evidence. Rehwald et al. (2018) show that traditional activation programs have little impact on employment outcomes for sick‐listed workers, while graded return‐to‐work programs show more promise. Similarly, Johansson and Palme (2005) find that reducing sick leave compensation significantly reduced both the incidence and duration of sick leave. Other studies highlight institutional factors: Markussen et al. (2011) show that absenteeism varies due to worker heterogeneity and doctor discretion, while Stearns and White (2018) find that paid sick leave mandates reduce overall absenteeism by preventing disease spread. Evidence from the Italian public sector also shows that reducing sick leave compensation and increasing monitoring reduced short‐term absenteeism but had little effect on long‐term absences De et al. (2014). Additionally, Fevang et al. (2014) document how short‐term employer liability for sick pay creates a “sick pay trap,” discouraging firms from reintegrating previously sick employees due to financial risk. Pichler (2015) find that sick leave follows a procyclical pattern, with fewer absences during economic downturns, suggesting that financial security influences absence behavior. Similarly, D'Amuri (2017) show that stricter monitoring policies reduce sick leave duration, indicating that administrative oversight can reduce moral hazard. Finally, Puhani and Sonderhof (2010) show that reducing sick pay lowers absences without harming health, Ziebarth and Karlsson (2014) confirm that expanding sick pay increases absenteeism without improving job readiness, and Pettersson‐Lidbom and Thoursie (2013) find that increasing cash benefits for short‐term sick leaves paradoxically reduced total absenteeism, likely due to incentives favoring short but more frequent absences.

This study extends the literature by showing that sick‐listed workers respond more to the prospect of mandatory activation than to the content of activation programs. It questions the cost‐effectiveness of such measures, suggesting that similar short‐term employment effects could be achieved simply by signaling stricter eligibility criteria—without the added expense of activation programs.

The remainder of this paper is organized as follows. In Section 2, I describe the institutional setting in Denmark and explain the elements of the reform. Following this, Section 3 lays out the empirical framework and the regression model employed for estimating the impact of the reform. Section 4 discusses the dataset used in this study, and Section 5 presents and discusses the results. Finally, in Section 6, I draw conclusions from the findings and discuss their implications.

## 2 Institutional Setting

In Denmark, several government programs are designed to provide financial support to individuals facing challenges in sustaining their employment due to health issues or those who have lost their jobs. Among the most important programs are paid sick leave, disability insurance, means‐tested social assistance, subsidized work and unemployment insurance benefits. Given the intricate interplay between paid sick leave and these other programs, a summary of each is helpful before we delve into the specifics of the paid sick leave program.

First, the disability insurance program is permanent, providing a consistent, high benefit until the recipient reaches pension eligibility age. It is tailored specifically for chronically ill individuals. To qualify for this benefit, applicants undergo a thorough assessment process. This entails comprehensive documentation of the individual's health status, living situation, ability to work, and efforts taken to enhance their work capacity.

Second, the unemployment insurance program in Denmark provides a buffer against job loss and operates on a voluntary membership basis. Facilitated by state‐sanctioned insurance funds termed “A‐kasser,” individuals contribute membership fees. The program offers substantial financial aid, approximating 19,000 DKK (about 2547 Euro) for those unemployed for up to two years. For benefit eligibility, individuals need to have been a member of an unemployment insurance fund for at least 1 year.

Third, the subsidized work program, known as “flex job”, is targeted at individuals whose work capabilities have been reduced due to long‐term illness or disability. As part of the subsidized work program, employers hire individuals for part‐time positions, with the obligation to pay only for the hours during which the individual is productive. This implies that if a person's health condition reduces their work capacity by 50%, the employer compensates only for those hours that are productive. The program further ensures the individuals' financial stability by supplementing their wages with a municipality‐provided transfer payment. This payment is approximately equivalent to the benefit level of unemployment benefits.

Lastly, the Danish social assistance program, being means‐tested, acts as a safety net for those ineligible for other types of social insurance. The benefit is modest, and individuals can only claim it if their wealth does not surpass 10,000 DKK (1500 euro). Additionally, in many instances, beneficiaries must participate in job activation programs.

Now, moving on to the specifics of the paid sick leave program. Paid sick leave in Denmark is a form of government‐provided financial support for people who are unable to work due to illness or injury. In order to qualify for paid sick leave, the person must provide a medical certificate from a doctor certifying their inability to work. Employed individuals are entitled to paid sick leave if they have worked at least 240 h within the past 6 months prior to the first day of absence and have been employed for at least 40 h in each of at least five of those months. Paid sick leave in Denmark is 19,728 DKK (2651 EURO). However, the employer is only entitled to sick pay reimbursement after 4 weeks of sick leave. For employed individuals, who make up roughly nine out of 10 cases, the amount is paid directly to the employer as salary compensation while the employee is on sick leave, although the employer remains obligated to pay the full wage during the absence. Unemployed individuals, who account for roughly one out of 10 cases, are also entitled to sick pay if they are eligible for unemployment benefits and become sick or injured during their unemployment period, thereby being exempt from the otherwise strict activation requirements. In contrast to employed individuals on paid sick leave, the sick pay amount in these cases is paid directly to the unemployed recipient as a transfer payment.

In the next section, I will discuss how the 2014 reform changed the paid leave system.

### 2.1 The 2014 Reform on Paid Sick Leave

In July 2014, a major reform was implemented in the Danish paid sick leave system. The core element of the reform was the introduction of an earlier reassessment point at 26 weeks, replacing the previous threshold at 56 weeks. At this reassessment point, municipalities must evaluate whether a person continues to meet the conditions for receiving paid sick leave. The evaluation is based on seven specific criteria introduced by the reform, which include: (1) ongoing or pending medical treatment, (2) awaiting clarification from a rehabilitation team, (3) having a life‐threatening illness, (4) being in the process of applying for disability pension, (5) needing a clarifying intervention to assess work capacity, (6) being involved in a work injury compensation case, or (7) undergoing medical investigation. At least one criterion must be met for the individual to retain eligibility for paid sick leave beyond 26 weeks.

If none of these criteria are fulfilled, the individual's sick leave spell is not extended. In such cases, the person is transferred to a mandatory activation program known as the job clarification program (jobafklaringsforløb). This program is designed to support return to work through intensified case management, activation activities, rehabilitation, and job search.

The reform also changed how benefits were paid. Individuals not meeting one of the seven criteria lost sickness benefit entitlement and entered the Job Clarification Program (JCP), where the benefit level is up to 40% lower than the sickness benefit rate of DKK 19,728. For the unemployed, this meant a direct cut in transfers, while for the employed it implied reduced employer reimbursement.

**FIGURE 1:**
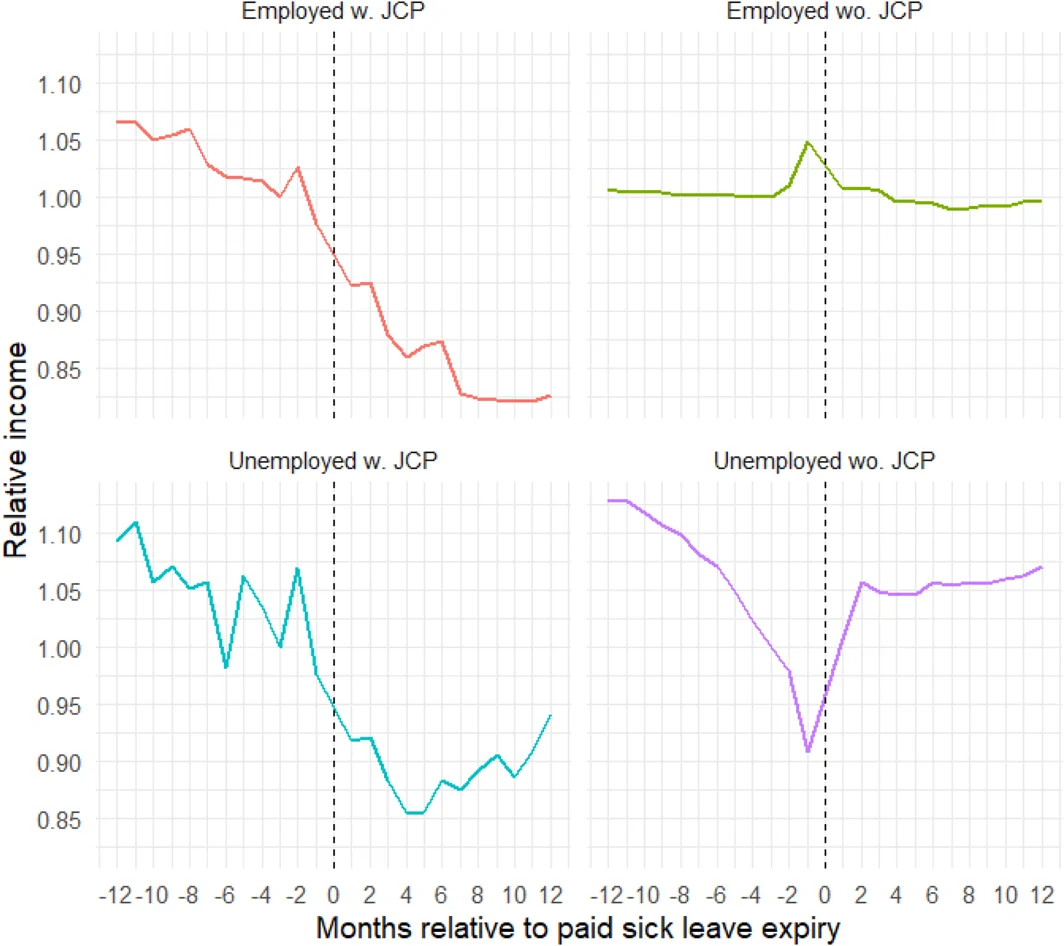
Relative gross income 12 months before and 12 months after paid sick leave expiry. The vertical dashed line marks the expiry of paid sick leave. Panels split individuals by employment status (employed vs. unemployed) and participation in the Job Clarification Program (JCP). Relative gross income is normalized to one 3 months prior to expiry.

To better understand this point, Figure 1 shows gross income before and after paid sick leave expiry, normalized to one 3 months prior. The figure separates individuals by employment status and JCP participation. Those not entering the JCP display relatively constant income profiles: employed individuals return to work with unchanged earnings at benefit expiry, while unemployed individuals transition to unemployment benefits at roughly the same level as sickness benefits at benefit expiry.

For employed individuals entering the JCP, income is not directly affected by the benefit cut, since the reduction applies only to the employer reimbursement. Instead, their income displays a gradual downward trend, likely reflecting declining wage payments as the sick leave spell lengthens. Thus, no sharp change occurs at the time of expiry, only a continuous decline.

For unemployed individuals entering the JCP, income is directly affected by the benefit reduction, since their transfer is lowered by up to 40%. This appears in the figure as a more discrete drop in gross income around the paid sick leave expiry at time 0.

To sum up, unemployed individuals exhausting their benefits may be affected by the reform both through a benefit cut when enrolling in the JCP and through increased activation requirements. Employed individuals, on the other hand, are only affected through increased activation. This will become relevant later when I disentangle the effects.

To enhance understanding of the reform's impact, Table 1 wraps up how the reform affected individuals on paid sick leave depending on when they enrolled in the paid sick leave program. I divide the individuals into two distinct groups, which will be referred to throughout this paper.

**TABLE 1:** Group, reassessment point and enrollment in job clarification program.

Group	Time period	Reass. Point	Enrollment in JCP
R56	Before January 2014	56 weeks	Not applicable
R26	From January 2014 onwards	26 weeks	Likely after 26 weeks

The first group, labeled “R56,” consists of individuals who enrolled in paid sick leave before January 2014. These individuals are reassessed at 56 weeks (R56), in accordance with the rules prior to the reform. At that time, the job clarification program did not exist, and individuals were either extended on sick leave or transitioned into other tracks such as the social assistance program. As a result, R56 individuals are not eligible for job clarification and do not enroll in that program. The second group, labeled “R26,” consists of individuals who enrolled in paid sick leave from January 2014 onwards, after the reform was implemented. They are reassessed at 26 weeks, and if they do not meet the extension criteria, they are likely to be transferred to the job clarification program. The reform thus moved the reassessment point forward from 56 to 26 weeks.

Because reassessment timing is determined solely by the sick leave start date, the reform creates quasi‐experimental variation in exposure to the new rules. I exploit this variation to estimate the reform's causal effects, as described in the next section's empirical framework.

## 3 Empirical Framework

In this section, I describe the empirical framework used to estimate the effect of the January 2014 reform on employment outcomes. The reform reduced the reassessment threshold for paid sick leave from 56 to 26 weeks, creating a sharp policy change that increases early exposure to mandatory activation. The focus is on how this policy affects the probability of returning to employment.

The identification strategy exploits the discontinuity in treatment assignment at the January 2014 cutoff using a Two‐Stage Least Squares (2SLS) Instrumental Variables (IV) approach within a Regression Discontinuity Design (RDD) framework. Individuals who enrolled in paid sick leave before January 2014 remained under the 56‐week reassessment rule, while those who enrolled in or after January 2014 were subject to the new 26‐week reassessment rule. This allows for a direct comparison of employment outcomes for individuals just before and after the cutoff. For the RDD estimation, I further restrict the full sample to individuals with paid sick leave durations longer than 25 weeks, ensuring that only those affected by the reassessment point are included. I refer to this as the restricted sample. In the data section, I provide additional details on how the sample is constructed.

More formally, let x denote the enrollment date into paid sick leave, which serves as the running variable. The treatment indicator $T_{i}$ is defined as:

(1) $$T_{i}=1\left(x\geq \text{January}\;2014\right)$$

where $T_{i}=1$ if individual i enrolled in paid sick leave on or after January 2014 and $T_{i}=0$ otherwise. The first stage of the 2SLS estimation uses $T_{i}$ as an instrument for a dummy indicating whether the person is enrolled in paid sick leave:

(2) $$S_{i}=\alpha +\delta T_{i}+f\left(x\right)+X_{i}^{\prime }\gamma +\nu_{i}$$

where $S_{i}$ is a binary variable equal to one if the individual is enrolled in paid sick leave and 0 otherwise. The second stage estimates the effect of predicted sick leave enrollment on employment:

(3) $$Y_{i}=\theta +\beta {\hat{S}}_{i}+f\left(x\right)+X_{i}^{\prime }\lambda +\epsilon_{i}$$

where $Y_{i}$ represents employment at different time horizons after enrollment, ${\hat{S}}_{i}$ is the predicted probability of sick leave enrollment from the first stage, f(x) is a function of the running variable to control for underlying time trends and, in alternative specifications, includes a second‐order polynomial in calendar time to account for seasonality, reflecting higher sick leave incidence in winter and lower incidence in summer, and $X_{i}$ is a vector of control variables which are age, gender, education, and pre‐enrollment income. The coefficient β captures the causal effect of paid sick leave enrollment on employment probability.

For the estimates to be valid, the assumptions underlying the RDD framework must hold. These assumptions are stated in Imbens and Thomas (2008), but I will briefly outline them here. First, the conditional expectation of potential outcomes must be smooth at the cutoff, meaning that in the absence of treatment, employment probability would have evolved continuously over time. Any observed discontinuity in employment outcomes at the threshold should therefore be attributable to the policy change. Second, individuals near the cutoff must be comparable, which requires that assignment to the treatment is locally as good as random. This assumption ensures that any difference in employment probability at the cutoff is not driven by pre‐existing differences between treated and untreated individuals. Third, there must be no precise manipulation of the running variable, meaning that individuals should not be able to systematically sort themselves into or out of treatment by selecting their enrollment date. If individuals could strategically delay or advance their enrollment to remain under the more lenient reassessment rule, the estimated effect may be biased. I will address these assumptions in the Regression Discontinuity section.

In the next section, I describe the dataset used for the analysis.

## 4 Data

This study utilizes panel data from administrative records provided by Statistics Denmark, encompassing the entire Danish population from 2008–2019. The data includes comprehensive information on income, employment status, benefits, education and demographic variables, as well as individual person IDs.

Income data are obtained from exact tax records, providing monthly information on individual income, including wage and income transfers. To determine the duration on paid sick leave, I use a database from the Danish Agency for Labor Market and Recruitment, providing weekly information for every person in Denmark regarding the benefits they receive. To attain information on education, I use data from the Ministry of Higher Education and Science, which contains yearly information on the highest completed education level for all Danish individuals. Finally, demographic information such as age, gender and ethnicity was obtained from the Danish Population Register. Using the administrative data, I construct the variables presented in Table 2.

**TABLE 2:** Data description for variables.

Variable	Description
Duration	Number of weeks on paid sick leave, starting from enrollment until switching to another benefit type or returning to work. A spell ends only after four consecutive weeks without sick leave benefit payments. This criterion helps to avoid including small spells where individuals may have taken a week off from their sick leave, for example, due to vacation.
Gross income	Total wage and transfer income in 2021 DKK prices, measured from the beginning of the sick leave spell and for 1‐5 subsequent years. The gross income is calculated annually (e.g., from the enrollment month to the same month in the following year for the first year, from the 13th to the 24th month for the second year, and so on).
Age	Age in years at the beginning of the paid sick leave spell.
Male	Dummy variable indicating if the person is male.
Native	Dummy variable indicating if the person is native Danish. A native is defined as having at least one Danish‐born parent with Danish citizenship, regardless of their own birthplace.
Uneducated	Dummy variable indicating if the person has no education by November 1 year prior to enrolling in the sick leave program.

The next subsection describes the construction of the samples.

### 4.1 Constructing of the Samples

To analyze the impact of the 2014 policy reform in Denmark, I construct two primary samples: R56 and R26. In both samples, I exclude individuals who died or emigrated within five years after enrolling in the paid sick leave program. This corresponds to a small share of about 4% in each sample. The first sample, R56, consists of individuals who enrolled in the sick leave program in the second half of 2013, before the reform. These individuals undergo a reassessment at the 56‐week mark and were unlikely to participate in the job clarification program, meaning they are considered untreated. The second sample, R26, includes individuals who enrolled in sick leave in the second half of 2014, after the reform was implemented. This group undergoes reassessment at 26 weeks and is likely to participate in the job clarification program at that point.

These two samples will serve as descriptive samples. I focus on the second half of each year solely for descriptive purposes, as there are pronounced seasonal effects with more new sick‐leave cases—particularly in January. By using the latter half of each year, these seasonal patterns are substantially reduced.

The two groups, R26 and R56, consist of 174,976 and 168,930 unique individuals, respectively. From this full sample, I also construct two restricted subsamples, likewise denoted R26 and R56, but limited to individuals with sick leave durations exceeding 25 weeks. These restricted samples ensure that the analysis focuses on those directly exposed to the reassessment at 26 weeks, which forms the basis for the RDD estimation. Note, this choice of sample could potentially involve endogeneity, meaning that individuals respond before the reassessment point. However, as we will see in the next sections, this does not appear to be the case. I return to this point later, where the survival curves likewise show no evidence of anticipatory behavior before week 26. Further, in the restricted samples, I also exclude individuals who either died or emigrated, corresponding to a small share of 6% in each sample.

Table 3 provides descriptive statistics for both the full and restricted samples of the two groups, based on individuals who enrolled in the second half of 2013 and 2014. For comparability, the sample of individuals with durations exceeding 25 weeks is also shown for the second half of each year. However, in the estimation section, I also present results where I apply broader bandwidths of one, two, and three years around the reform cutoff, rather than restricting the analysis to the second half of 2013 and 2014 only.

**TABLE 3:** Descriptive statistics of the two sample groups (full and restricted samples).

	Full sample				Restricted sample (25+ weeks)			
	R26		R56		R26		R56	
Variable	Mean	Std. Dev.	Mean	Std. Dev.	Mean	Std. Dev.	Mean	Std. Dev.
Age	41.41	11.85	41.13	11.78	43.87	10.83	43.57	10.77
Male	0.44	0.50	0.45	0.50	0.38	0.48	0.38	0.49
Native born	0.90	0.30	0.90	0.30	0.89	0.32	0.89	0.31
No education	0.33	0.47	0.34	0.47	0.31	0.46	0.33	0.47
Prior gross inc. (DKK)	302,288	147,536	301,835	144,404	319,905	146,463	317,755	139,443
N	174,976		168,930		28,783		28,916	

*Note:* The table reports means and standard deviations. In the full sample, 83% for both R26 and R56 groups leave sick leave within 25 weeks. By construction, the restricted sample includes only spells longer than 25 weeks.

*Source:* Statistics Denmark and own calculations.

The mean age across the groups is quite similar in both samples: 41.41 years for “R26″ and 41.13 years for “R56″ in the full sample, and 43.87 and 43.57 years, respectively, in the restricted sample. Likewise, the gender proportions are comparable, with male shares of 0.44 and 0.45 in the full sample, and 0.38 in both groups in the restricted sample. Slight differences in education levels are observed, but these are small across both samples, with the share of individuals with no education being 0.33 and 0.34 in the full sample, and 0.31 and 0.33 in the restricted sample for “R26″ and “R56,” respectively.

Another important factor considered in this study is gross income 1 year prior to enrollment in paid sick leave. The values are highly similar across the two groups: 302,288 DKK (40,650 Euro) for “R26″ and 301,835 DKK (40,600 Euro) for “R56″ in the full sample, and 319,905 DKK and 317,755 DKK in the restricted sample. This further highlights the close balance between groups. In the full sample, 83% of individuals in both groups leave sick leave within 25 weeks, while, by construction, the restricted sample includes only longer spells.

Having presented the details of the samples constructed to evaluate the impact of the 2014 policy reform and having highlighted the balanced nature of these groups in terms of demographics and gross income, I now delve into the results.

## 5 Results

This section presents the key results of my analysis. The first subsection provides a visual inspection of the reform using the full sample, while the second subsection conducts a formal regression discontinuity analysis on those affected by the policy, that is, individuals with a duration of more than 25 weeks.

### 5.1 Visual Inspection of the Impact of the Reform

**FIGURE 2:**
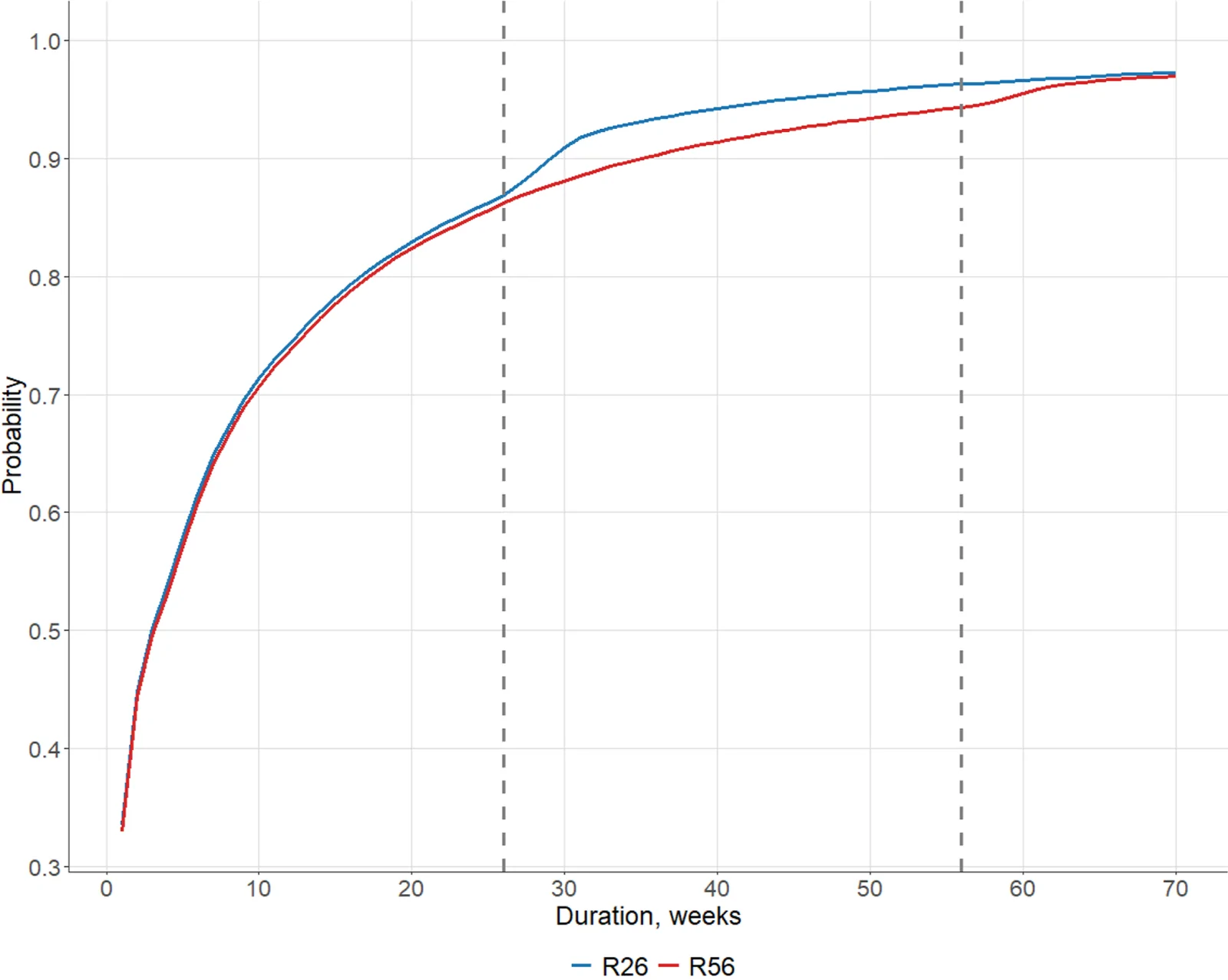
CDF of Paid Sick Leave Duration for Groups R26 and R56. The figure shows the CDF for two groups in the paid sick leave program. The “R26” group sees a significant exit increase at 26 weeks, with 87% exiting by this point and 92% by 31 weeks. The “R56” group has a similar exit rate at 26 weeks, but only 88% have exited by 31 weeks.

Figure 2 presents the cumulative distribution function (CDF) for the distinct groups of individuals on sick leave, each with a predefined reassessment point: group “R26” at 26 weeks and group “R56” at 56 weeks. In the “R26” group, a marked increase in the CDF is evident immediately after the 26‐week reassessment (highlighted by the first vertical line); at this point, 87% of individuals have exited the program, rising further to 92% by week 31. In contrast, for the “R56” group, approximately the same fraction has exited by week 26, though only 88% have exited by week 31.

Interestingly, the data show no significant increase in exits just prior to the reassessment dates, which would have been indicative of an anticipation effect. Instead, exits occur sharply at the reassessment points themselves, suggesting that individuals are responding to the reassessment decision rather than timing their exits in advance. This is consistent with the institutional setup, where individuals are only informed about the expiry of their benefits during the reassessment meeting itself and have no knowledge of it beforehand.

The figure also indicates that the majority of people are not affected by the earlier reassessment point, as 87% have already exited the program before this point.

**FIGURE 3:**
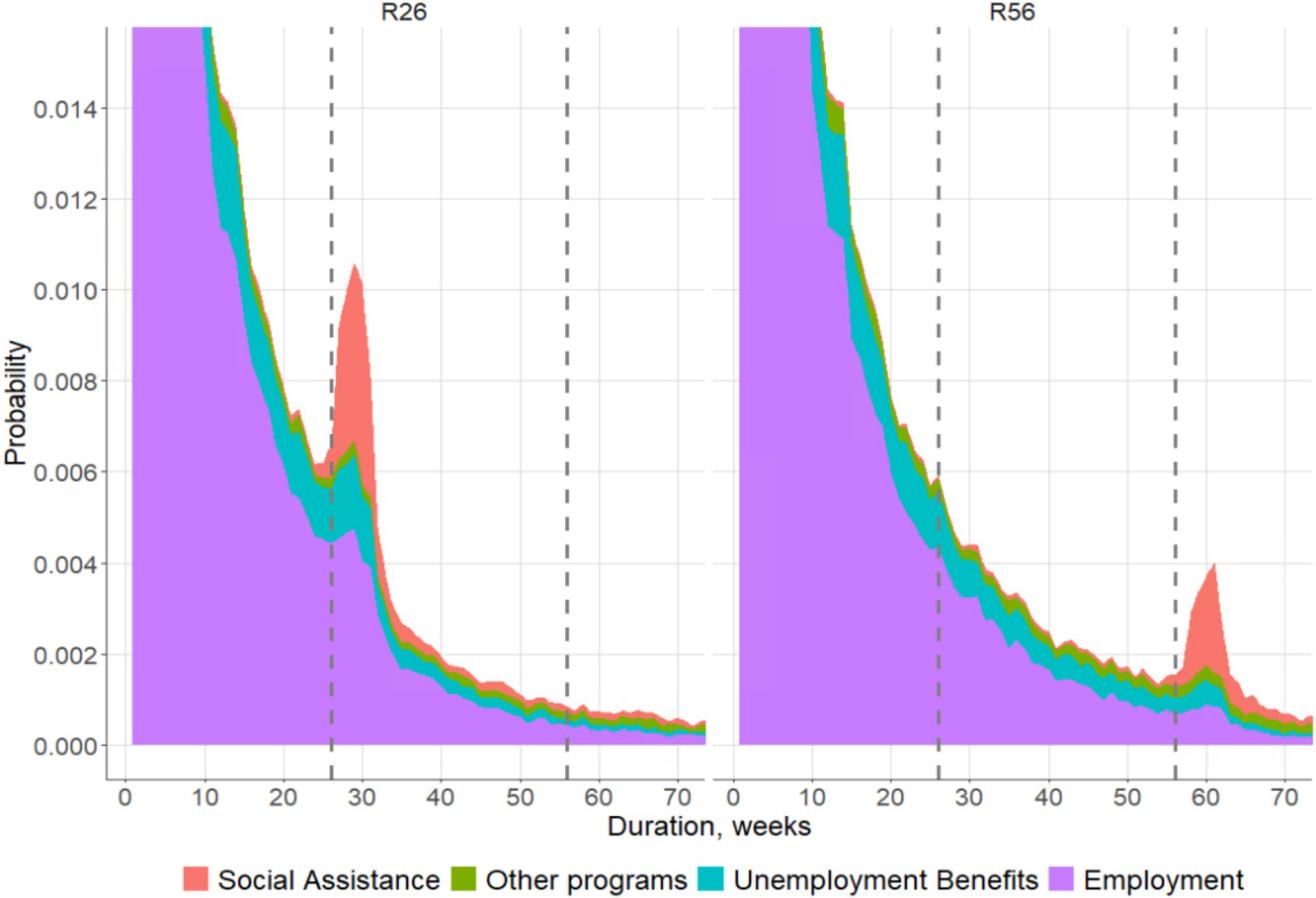
PDF of Paid Sick Leave Duration for Groups R26 and R56 by Benefit Type. The figure shows the PDF of weekly exits from paid sick leave in groups R26 and R56, categorized by exit destination.

To further examine exits from paid sick leave, Figure 3 displays the probability distribution function (PDF). The area under the curve illustrates the destinations of beneficiary exits. There are four main categories: “employment,” “unemployment benefits,” “social assistance,” and “other programs.” To maintain clarity, I refer to the third category as social assistance, rather than just the job clarification program, because it includes both the job clarification program and the standard social assistance program. However, 96% of this category consists of the job clarification program, while only 4% falls under the standard social assistance program. The “other programs” category includes programs such as disability insurance and subsidized work programs.

Prior to the reassessment point, the primary reason for exiting paid sick leave is a return to employment, with a smaller fraction of exits to unemployment insurance. Both groups exhibit the same exit pattern up to the reassessment point.

After the 26‐week point, a rise in the probability of exiting the program is observed, similar to the trend shown in the previous figure. This increase is driven by a rise in exits to employment, a small increase in exits to unemployment benefits, but more importantly, a notable increase in exits to the social assistance program.

**FIGURE 4:**
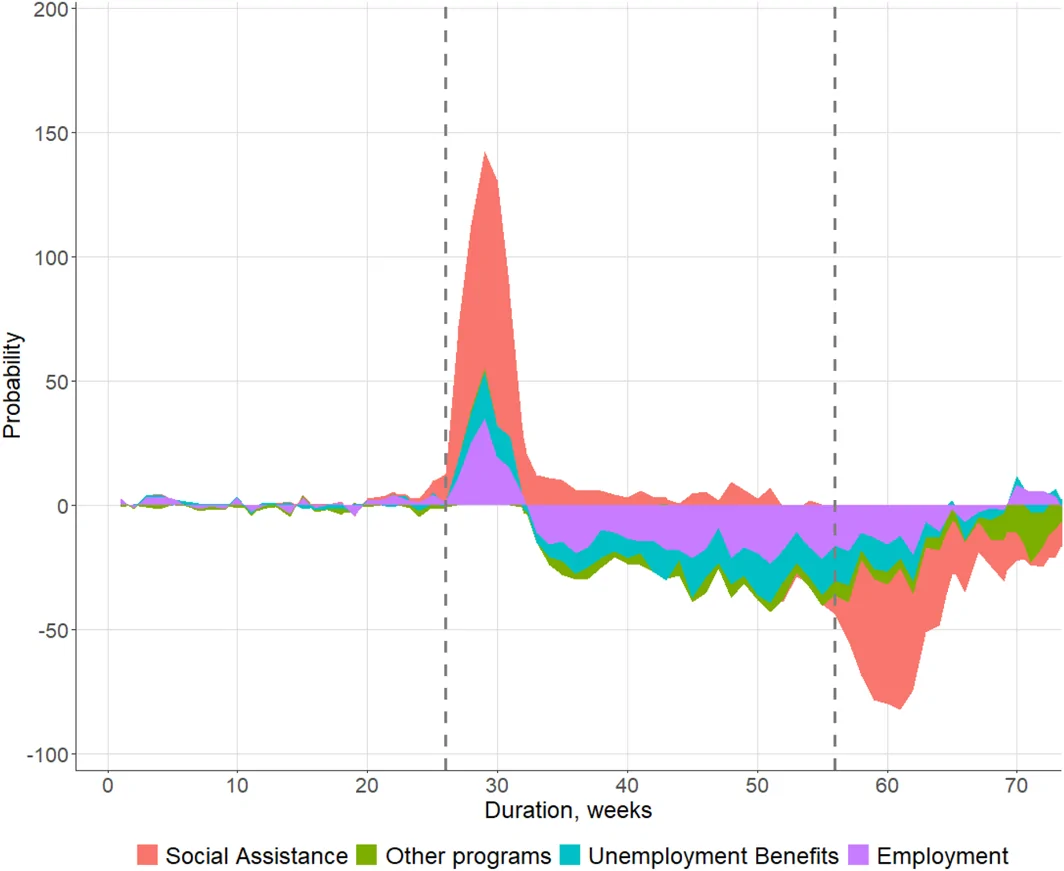
Change in Probabilities from Paid Sick Leave for Group R26 Compared to Group R56. This figure displays the relative increase in probabilities from paid sick leave for the R26 group compared to the R56 group, categorized by exit destination. Following the reassessment point at 26 weeks, the exit probability for R26 rises to 142% above that of R56, primarily due to increased transitions to social assistance, with smaller contributions from employment and other categories.

To further clarify the factors driving the spike in exits around the reassessment point, I calculate the change in the probability for each duration from R56 to R26 and assess the contribution of each of the four benefit categories, representing the relative change between the two groups shown in Figure 4. Prior to the reassessment point at 26 weeks, there is no difference in the probabilities of exiting paid sick leave in a given week. Immediately after 26 weeks, however, the probability of exiting increases, making it 142% more likely for the R26 group compared to the R56 group to leave paid sick leave by week 29. Of this 142% increase, 35% points are due to increased transitions to employment, 87% points to social assistance (primarily the job clarification program), 19% points to unemployment benefits, and 2% points to “other programs.” This indicates that the largest contribution to the increase around the reassessment point comes from the rise in social assistance, suggesting that individuals are largely transitioning to the job clarification program rather than unemployment benefits. Interestingly, the excess employment effect only emerges close to the reassessment point. This suggests that individuals may seek to return to work to avoid entering the program, but it could also be partially driven by the reduction in benefits. I will return to this issue later.

What is even more apparent from the graph is that the contribution of employment exits turns negative after 32 weeks, meaning that the rate of exits to employment was higher under the previous scheme. From week 32 onward, there is consistently a lower rate of exit to employment. This is likely due to a selection effect, where individuals with relatively better health are transitioning into the social assistance program instead of naturally returning to work from paid sick leave.

Put differently, this finding indicates that the reassessment point at 26 weeks seems to divert individuals who might have otherwise returned to employment into social assistance instead.

To conclude, the analysis highlights a significant shift in exit probabilities from paid sick leave around the reassessment point at 26 weeks. The spike in exits is primarily driven by transitions to social assistance, with more modest effects on employment exits. Notably, after week 32, the rate of exits to employment under the new scheme is consistently lower than under the previous scheme. This suggests that while the reassessment point prompts a redistribution of individuals to different benefits, it does so at the expense of natural returns to employment. Furthermore, the observed employment effect around the reassessment point suggests either a response to the benefit reduction or a threat effect, where individuals return to work early to avoid program enrollment.

### 5.2 Regression Discontinuity Design

In this section, I formally estimate the causal effect of the reform using a regression discontinuity design. I assess the validity of the identification strategy in Appendix A.1 and Appendix A.2, showing that the two groups are balanced with no evidence of selection. This analysis focuses on individuals with sick leave durations longer than 25 weeks to capture those directly affected by the policy. In Appendix A.5, I elaborate on this point by including individuals with shorter durations.

#### 5.2.1 First‐Stage: Effect on Paid Sick Leave Enrollment

**FIGURE 5:**
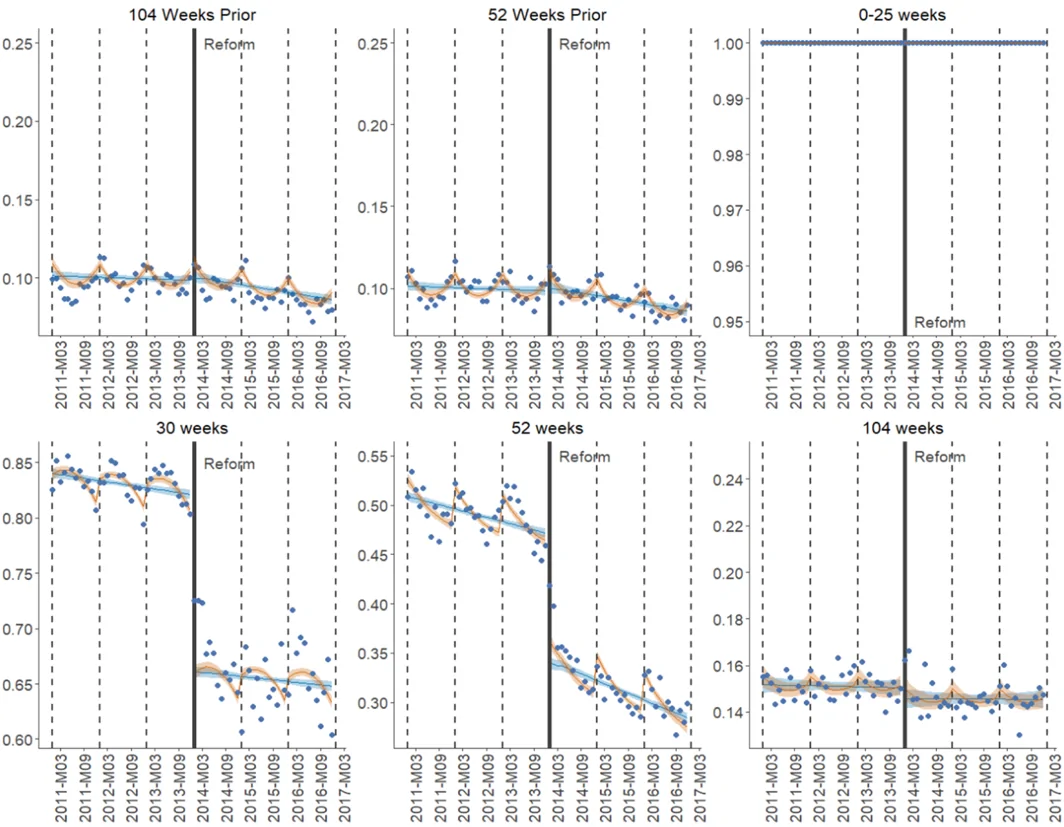
Probability of Being on Paid Sick Leave at Different Time Periods Before and After Enrollment, With (orange) and Without (blue) Seasonal Adjustment. This figure shows the probability of being on paid sick leave for individuals with spells longer than 25 weeks by enrollment month. Each panel represents a time period relative to enrollment: 104 weeks before, 52 weeks before, 0–25 weeks after, 30 weeks after, 52 weeks after, and 104 weeks after enrollment. The blue line shows a linear specification without seasonal adjustment, while the orange line shows a seasonally adjusted specification controlling for seasonality using a second‐order polynomial in calendar time. Shaded areas indicate confidence intervals around the fitted lines. The figure shows no differences across the reform prior to enrollment, while the reform induces a clear discontinuity in paid sick leave enrollment 30 and 52 weeks after enrollment.

Figure 5 shows the probability of enrollment into paid sick leave over time for individuals with a paid sick leave duration exceeding 25 weeks. The panels represent different time periods relative to enrollment: 104 weeks prior to enrollment, 52 weeks prior to enrollment, 0–25 weeks after enrollment, 30 weeks after enrollment, 52 weeks after enrollment and 104 weeks after enrollment. The figure also shows a linear trend (blue) and a seasonally adjusted model (orange), both estimated separately before and after the reform using individual‐level data. Shaded areas indicate confidence intervals around the fitted lines. The seasonally adjusted specification is included because the data exhibit a pronounced seasonal pattern, with a high incidence of sick leave in January followed by a gradual decline over the year. With a wide bandwidth, this seasonality is largely smoothed out in the linear fit, whereas with a narrower bandwidth the linear trend tends to overfit the seasonal pattern rather than capture the underlying discontinuity. I return to this issue later.

At 104 weeks prior to enrolling in paid sick leave, approximately 10% of individuals are experiencing another spell of paid sick leave. Importantly, there is no noticeable difference in the proportion of individuals enrolled in paid sick leave 104 weeks before enrollment when comparing groups across the reform. A similar pattern is observed in the figure showing 52 weeks prior to enrollment, indicating that no selection effects are present, as expected.

By definition, because the sample is conditioned on individuals having a spell lasting longer than 25 weeks, the upper‐right panel shows that 100% of individuals in the sample are enrolled in paid sick leave during the period between 0 and 25 weeks of their spell.

The lower panels show a shift in the probability of enrolling in paid sick leave for individuals enrolling just after the reform compared to those enrolling before the reform. Specifically, 30 weeks after enrollment into paid sick leave for those with a duration of more than 25 weeks, the probability of still being enrolled is about 65%, compared to about 82% for those enrolling before the reform.

This difference reflects the behavior of the complier group, that is individuals whose enrollment status responds to the reform by leaving paid sick leave earlier than they would have under the pre‐reform rules. Appendix A.3 shows that this group either transitions directly into employment or, to a larger extent, is reallocated into the long‐term job clarification program. The appendix further documents that individuals entering the job clarification program are initially largely passive but gradually transition toward activation measures and employment over time.

Thus, the reform clearly exogenously shifts the probability of being enrolled in paid sick leave, as was also demonstrated in the previous section. Even after 52 weeks, the probability of being enrolled in paid sick leave remains approximately 20% points lower after the reform. Interestingly, after 104 weeks, there is no difference before and after the reform, which is because the old assessment point at 56 weeks forces individuals out of the paid sick leave program. This effect was also visible in Figure 4.

Table 4 summarizes the estimated discontinuity in the probability of being on paid sick leave 30 weeks after enrollment, that is the first‐stage effect of the 2014 reform. The estimates correspond to the drop observed in Figure 5 at 30 weeks, while additionally controlling for age, gender, being native‐born, and education. Estimates are reported across different bandwidths, polynomial orders, and kernel functions, and are shown both without seasonal adjustment and with seasonal adjustment, where the latter controls for seasonal effects using a second‐order polynomial in calendar time, as shown in Figure 5.

**TABLE 4:** First‐stage estimates with and without seasonal adjustment.

	Without season			With season		
	Coef.	*t*	*F*	Coef.	*t*	*F*
Bandwidth						
12 months	−0.073	−4.81	23.15	0.343	40.73	1658.84
24 months	−0.108	−11.66	136.06	−0.124	−16.25	264.12
36 months	−0.130	−18.38	337.60	−0.141	−22.66	513.61
Polynomial order						
Linear	−0.130	−18.38	337.60	−0.141	−22.66	513.61
Quadratic	−0.093	−8.84	78.06	−0.109	−12.77	163.12
Cubic	−0.059	−5.92	35.03	−0.075	−8.18	66.83
Kernel function						
Triangular	−0.130	−18.38	337.60	−0.141	−22.66	513.61
Uniform	−0.139	−24.11	581.49	−0.149	−26.19	685.90
Epanechnikov	−0.136	−19.99	399.42	−0.144	−23.63	558.57

*Note:* The table reports first‐stage estimates of the effect of the 2014 reform on the probability of being on paid sick leave 30 weeks after enrollment. The left panel reports specifications without seasonal adjustment, while the right panel reports specifications with seasonal adjustment. All models control for age, gender, native status, and education. Based on 143,819 observations.

Across specifications, the estimates with and without seasonal adjustment are very similar and lead to the same qualitative conclusions. In all cases, the coefficient is negative and statistically highly significant, confirming that the reform exogenously reduced the probability of remaining on paid sick leave among individuals with spells longer than 25 weeks. The magnitude of the effect increases slightly with larger bandwidths, reflecting greater precision when including more observations, and the F‐statistics are far above the conventional threshold of 10 in all specifications, indicating a strong first stage.

An exception arises for the narrowest bandwidth of 12 months in the season‐adjusted specification, where the estimate differs markedly from the remaining results. This reflects the difficulty of separately identifying seasonal effects and the underlying time trend within a very narrow bandwidth, as limited calendar‐time variation makes the seasonal component poorly identified. For wider bandwidths, the season‐adjusted estimates are stable.

#### 5.2.2 Second‐Stage: Effect on Employment After Reassessment

Table 5 presents the second‐stage estimates from the fuzzy RDD, reported both without seasonal adjustment and with seasonal adjustment using a second‐order polynomial in calendar time. The estimates capture the causal effect of remaining on paid sick leave 30 weeks after enrollment on the probability of being employed immediately after the reassessment point. Importantly, this employment effect reflects only the immediate, direct impact of the reform at the reassessment threshold and does not capture potential longer‐term employment effects arising from participation in the job clarification program or subsequent activation measures.

**TABLE 5:** Second‐stage IV estimates of the effect of paid sick leave at 30 Weeks on employment, with and without seasonal adjustment.

	Without Season		With Season	
	Coef.	*t*	Coef.	*t*
Bandwidth				
12 months	0.126	1.58	−0.731	−75.74
24 months	−0.097	−3.26	−0.216	−9.26
36 months	−0.141	−7.22	−0.208	−12.25
Polynomial order				
Linear	−0.141	−7.22	−0.208	−12.25
Quadratic	−0.052	−1.21	−0.226	−7.15
Cubic	0.197	1.73	−0.247	−4.10
Kernel function				
Triangular	−0.141	−7.22	−0.208	−12.25
Uniform	−0.137	−8.28	−0.201	−13.69
Epanechnikov	−0.151	−8.41	−0.206	−12.88

*Note:* The table reports second‐stage instrumental variable estimates from the fuzzy regression discontinuity design. The left panel reports specifications without seasonal adjustment, while the right panel reports specifications with seasonal adjustment using a second‐order polynomial in calendar time. The estimates capture the causal effect of remaining on paid sick leave 30 weeks after enrollment on the probability of employment measured immediately after the reassessment point, using the 2014 reform as an instrument. All specifications are based on 143,819 observations.

For specifications without seasonal adjustment, the choice of bandwidth is crucial. With a 36‐month bandwidth, a linear functional form effectively smooths out seasonal fluctuations, yielding stable estimates. In contrast, with shorter bandwidths, the linear specification partly captures seasonal patterns rather than the underlying time trend, leading to greater sensitivity of the estimates to bandwidth choice.

Examining different polynomial orders further illustrates this issue. In the absence of seasonal controls, higher‐order polynomials tend to overfit the data: quadratic and cubic specifications pick up seasonal variation that is unrelated to the underlying time trend around the cutoff. This overfitting explains the substantial variation in coefficients across polynomial orders in the non‐season‐adjusted specifications.

By contrast, the estimates from the season‐adjusted models are highly stable across bandwidths, polynomial orders, and kernel choices. The only notable exception is the narrowest bandwidth of 12 months, where it is not possible to separately identify the underlying time trend and seasonal effects due to limited calendar‐time variation. For wider bandwidths, the season‐adjusted coefficients are consistent and robust.

In terms of magnitude, the linear specification with a 36‐month bandwidth implies that remaining on paid sick leave 30 weeks after enrollment reduces the probability of returning to employment by about 14% points in the model without seasonal adjustment. When explicitly controlling for seasonal effects, the corresponding estimate increases to around 20% points. These estimates should be interpreted as local average treatment effects for individuals whose program status changed as a result of the reform.

**FIGURE 6:**
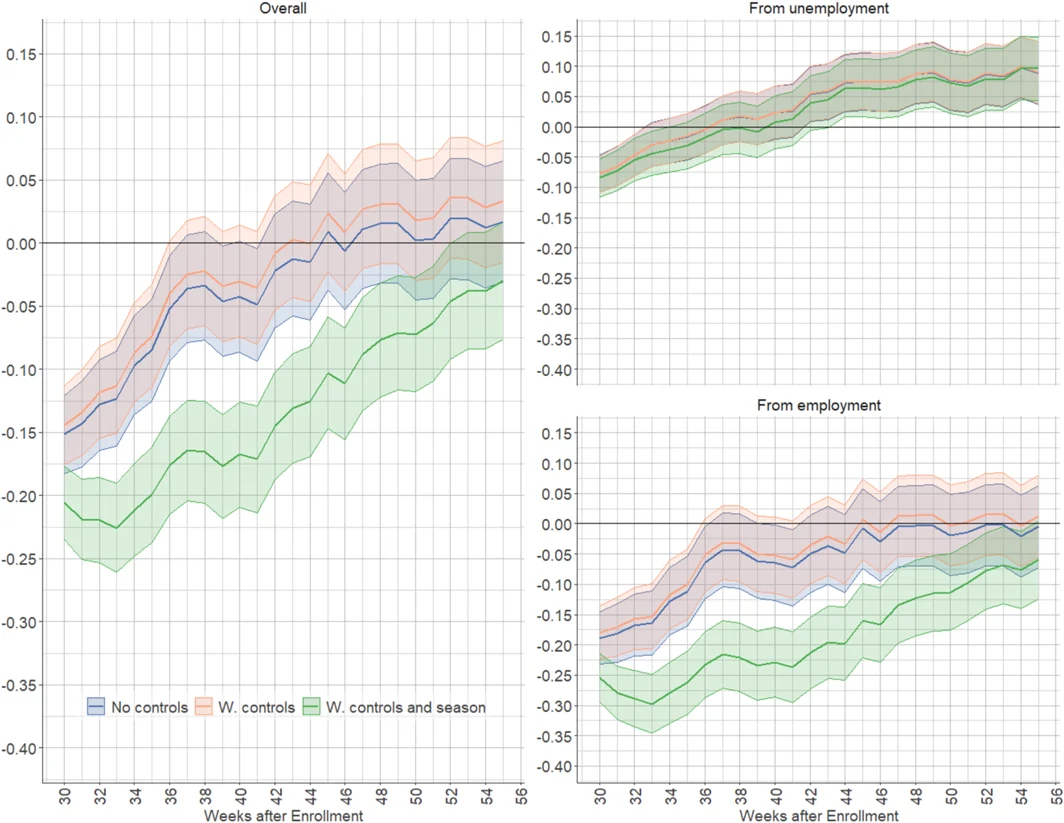
Estimated Effects of the Reform on Employment Probability. This figure presents the estimated effects of the reform on employment probability. The left panel shows the overall effect, including a seasonally adjusted specification, while the right panels disaggregate the results by prior labor market status (individuals coming from employment and unemployment, respectively). The *x*‐axis represents weeks since enrollment, and the *y*‐axis measures the probability of employment. Shaded areas indicate 95% confidence intervals.

While Table 5 captures the short‐term impact around the reassessment point, a longer horizon is required to assess whether the program activities have a persistent effect on employment prospects. For completeness, Appendix A.4 presents the corresponding reduced‐form effect on employment.Figure 6 therefore presents the second‐stage estimates from week 30–55 after enrollment. To ensure the robustness of these estimates, I employ a bandwidth of 36 months. This choice is deliberate; while the standard MSE‐based bandwidth selector (Calonico et al. 2014) suggests a window of less than 12 months, such a narrow interval is inappropriate here. It fails to encompass a full calendar year on each side of the cutoff, which is necessary to separately identify seasonal components from the underlying time polynomial. While shorter bandwidths yield similar results in seasonally adjusted models, the linear model remains sensitive to this specification.

The left panel of Figure 6 displays the overall estimated effects. At week 30, the effect is −14% points, identical to the 36‐week bandwidth estimate in Table 5. However, when adjusting for seasonal effects, the initial impact is even more pronounced at −21% points. This effect gradually declines over time and becomes statistically insignificant after roughly a year, suggesting that the reform's impact on employment probability is transitory.

To disentangle whether these results are driven by the reduction in benefits or a “threat effect” from activation measures, the analysis disaggregates the results by prior labor market status. Recall that for individuals coming from employment, the benefit reduction does not apply directly to the worker, as benefits are paid to the employer as compensation. Consequently, these individuals continue to receive their contractual wage and are primarily affected by the risk of activation. As shown in the right panels of Figure 6, the short‐term drop in employment probability remains clearly present for the “From employment” group. This strongly suggests that the observed reform effect is primarily driven by a threat effect—where individuals return to work to avoid program participation—rather than by the economic pressure of reduced benefits.

In contrast, the effect for individuals coming from unemployment is notably smaller, characterized by a minor negative short‐run effect followed by a small positive effect in the longer run. Overall, the findings indicate that once the initial threat effect vanishes after approximately a year, no statistically significant impact remains. Furthermore, the results suggest that individuals entering from unemployment may have been better off under the pre‐reform regime, where they benefited from a longer duration of paid sick leave without the immediate pressure of the new reassessment criteria.

## 6 Conclusion

This study examines the effects of a 2014 reform that reduced the duration of paid sick leave and introduced an activation program. The findings highlight that individuals on paid sick leave respond to policy changes primarily through a ‘threat effect’: a temporary increase in employment due to the risk of being transferred to an activation program. However, this effect appears to diminish over time, and there seems to be no sustained improvement in employment outcomes in the long run.

Instead of enhancing return‐to‐work rates, the reform appears to have led to a redistribution of individuals across different benefit schemes. Many individuals who might have stayed on paid sick leave longer instead transitioned into the job clarification program.

The key finding of this study is that it is not the activation program itself that improves employment outcomes but rather the threat of activation. The policy's main effect is to push individuals to return to work around the reassessment point, likely in response to the upcoming transition into the activation program, rather than well in advance of it, thereby avoiding the program altogether. This is in contrast to the intended purpose of activation programs, which are designed to improve employment readiness and facilitate a long‐term return to work. The results indicate that once individuals enter the program, their employment prospects do not improve relative to those under the previous system. This suggests that the activation program acts more as a punishment than as an effective policy tool.

This distinction is critical for understanding the mechanisms behind labor market effects of sick leave reforms. If activation programs do not improve job readiness, their cost‐effectiveness must be questioned. The results imply that policymakers could achieve similar short‐term employment gains simply by signaling stricter eligibility criteria, without incurring the additional costs associated with mandatory activation. However, the long‐term consequences of such a strategy remain uncertain. If individuals forced into early labor market reintegration are placed in poor job matches or return to work before full recovery, the initial employment gains may be offset by increased job instability or higher rates of future sick leave.

Overall, these findings highlight the limitations of applying standard unemployment insurance activation policies to sick‐listed individuals. Unlike the unemployed, whose job search behavior can be directly influenced by activation requirements, sick‐listed individuals face health‐related constraints that may not be easily altered through policy interventions. The fact that the threat of activation drives behavior, while the program itself does not improve employment outcomes, raises concerns about the design of sick leave policies. Future reforms should carefully balance the need for financial sustainability with the long‐term well‐being of sick‐listed workers, ensuring that policy changes do not merely shift costs from one program to another without meaningful improvements in employment outcomes.

## Conflicts of Interest

The author declares no conflicts of interest.

## Figures and Tables

**FIGURE A1:**
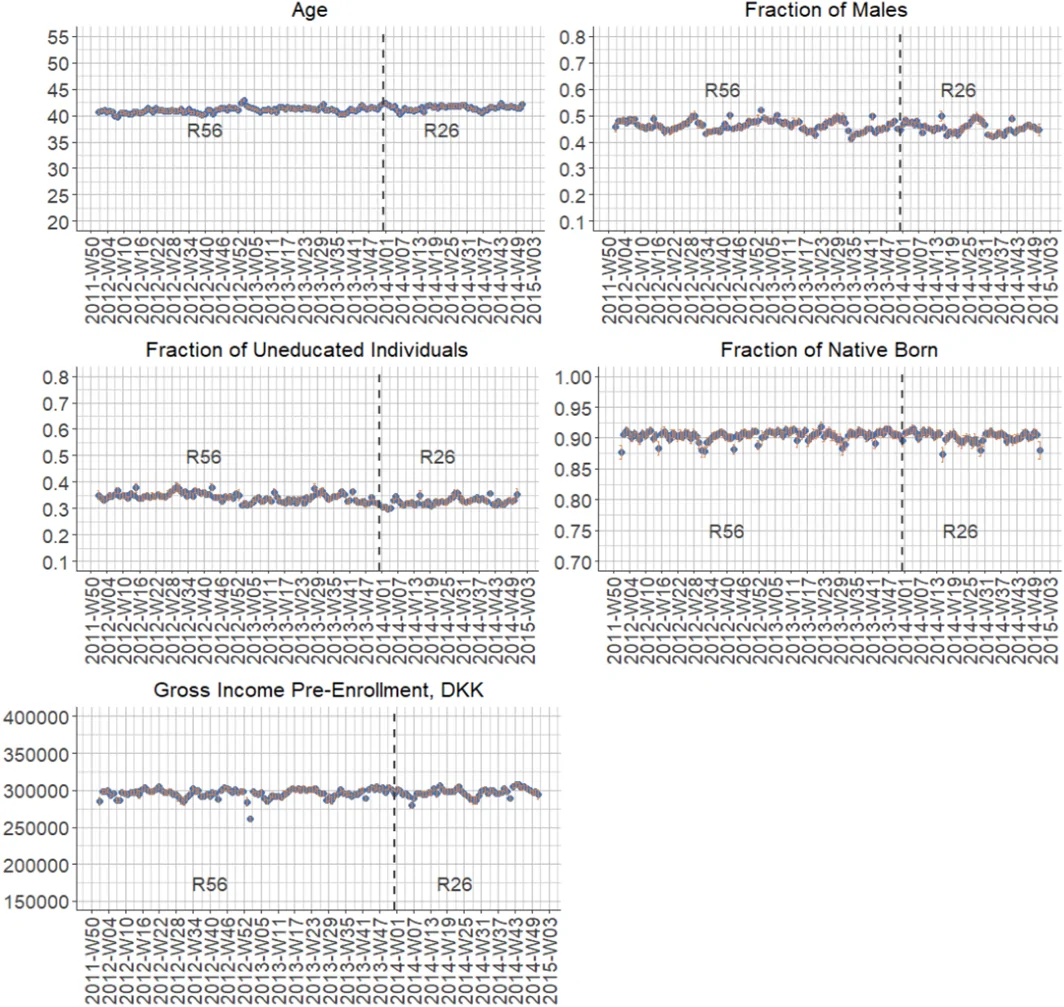
Balancing of Explanatory Variables for Paid Sick Leave Enrollment Full Sample. The figure displays the mean values of various variables for individuals enrolling in the paid sick leave program at different points in time. The graph shows no significant changes in the distribution of the variables over the two groups R56 and R26.

**FIGURE A2:**
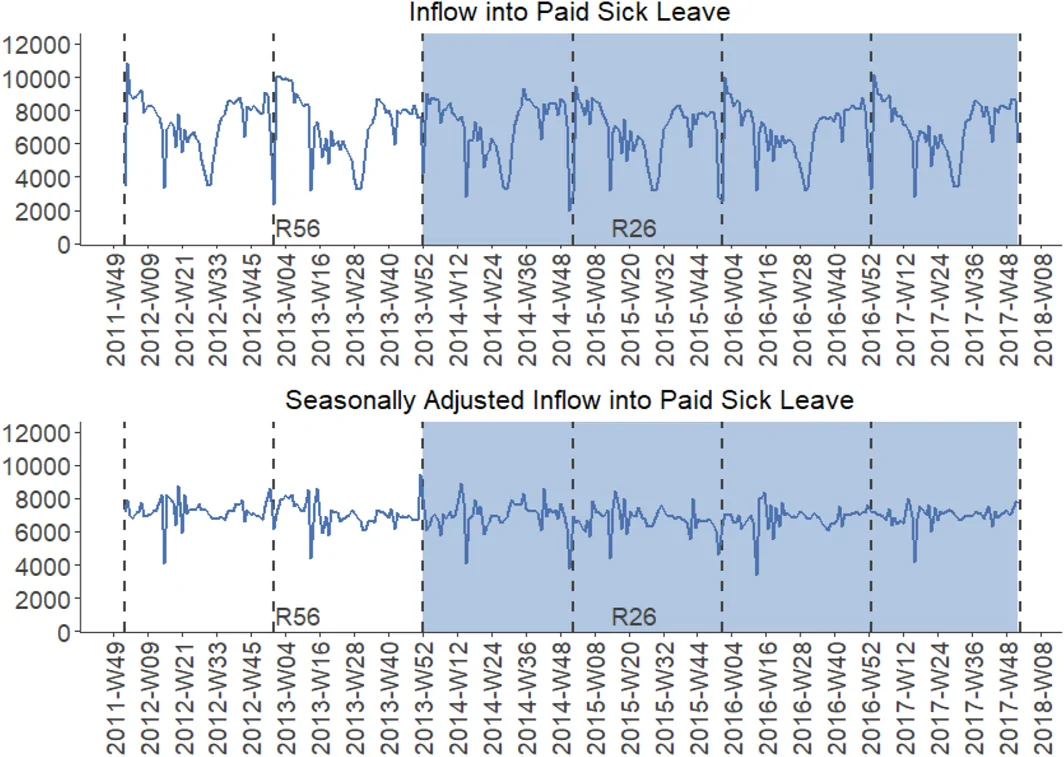
Weekly Inflow into Paid Sick Leave: Seasonally Adjusted and Unadjusted. The figure shows both the raw (unadjusted) inflow into paid sick leave (top panel) and the seasonally adjusted inflow (bottom panel). The shaded regions indicate periods of policy changes. The seasonally adjusted data reveals the underlying trend by removing the seasonal effects.

**FIGURE A3:**
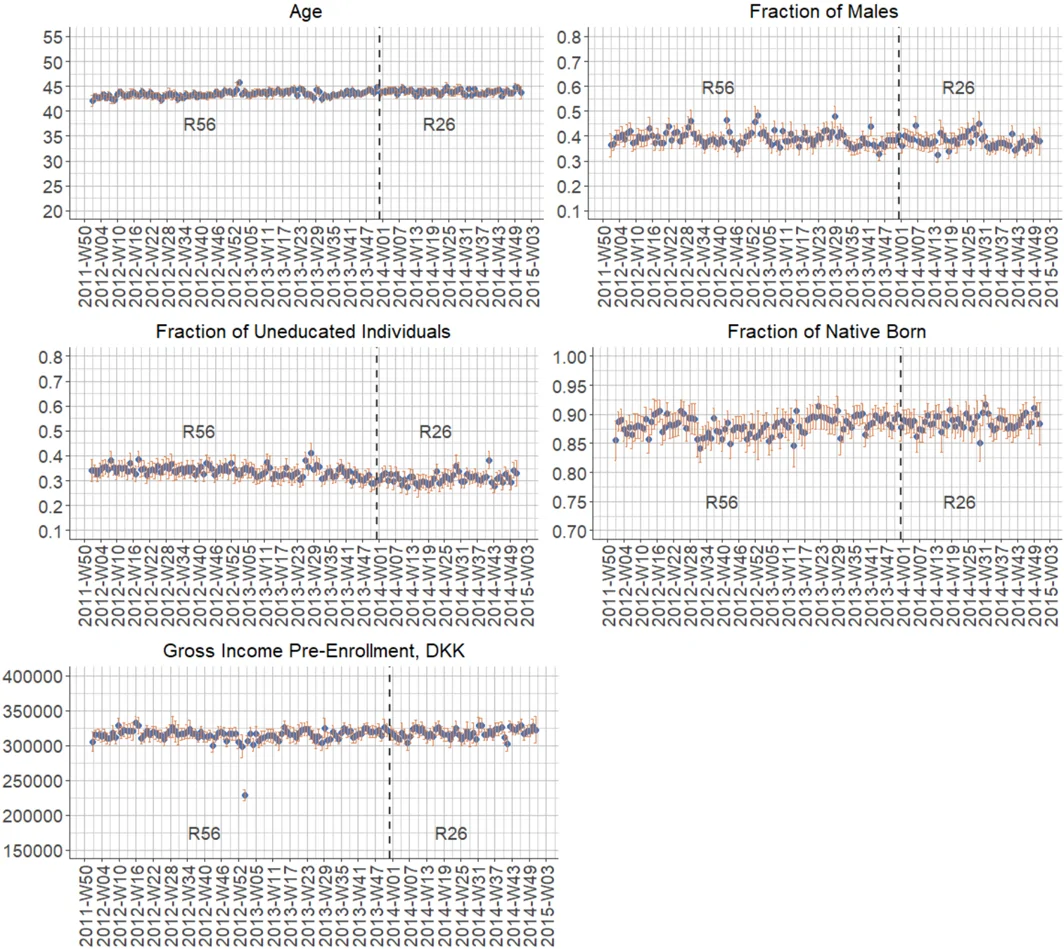
Balancing of Explanatory Variables for Individuals with Duration of More than 25 Weeks. This figure displays the mean values of various variables for individuals with sick leave durations exceeding 25 weeks, enrolled in the program at different points in time. The graph shows no significant changes in the distribution of the variables over the two groups R56 and R26.

**FIGURE A4:**
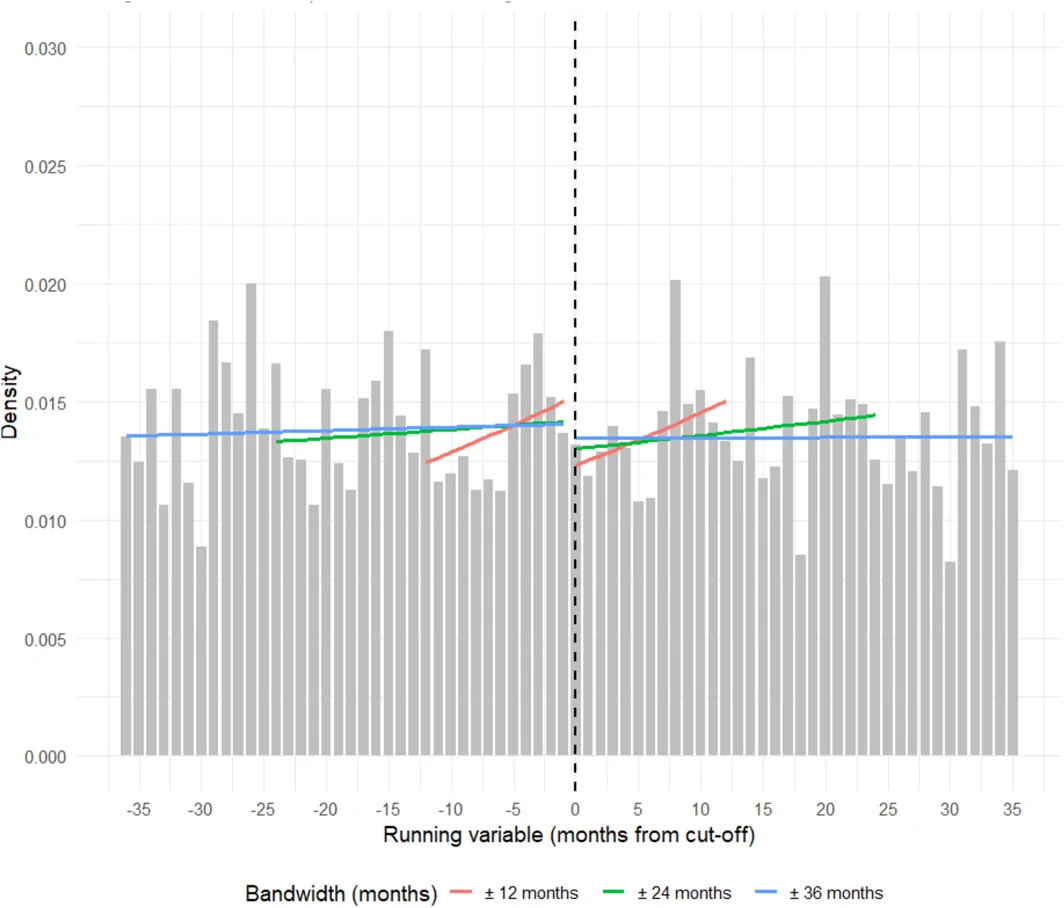
Density of the Running Variable Around the Reform Cut‐off. The figure plots the density of the running variable (measured in months from the reform cut‐off). Gray bars show the distribution of observations, and the colored lines show local linear fits within bandwidths of ±12, ±24, and ±36 months.

**FIGURE A5:**
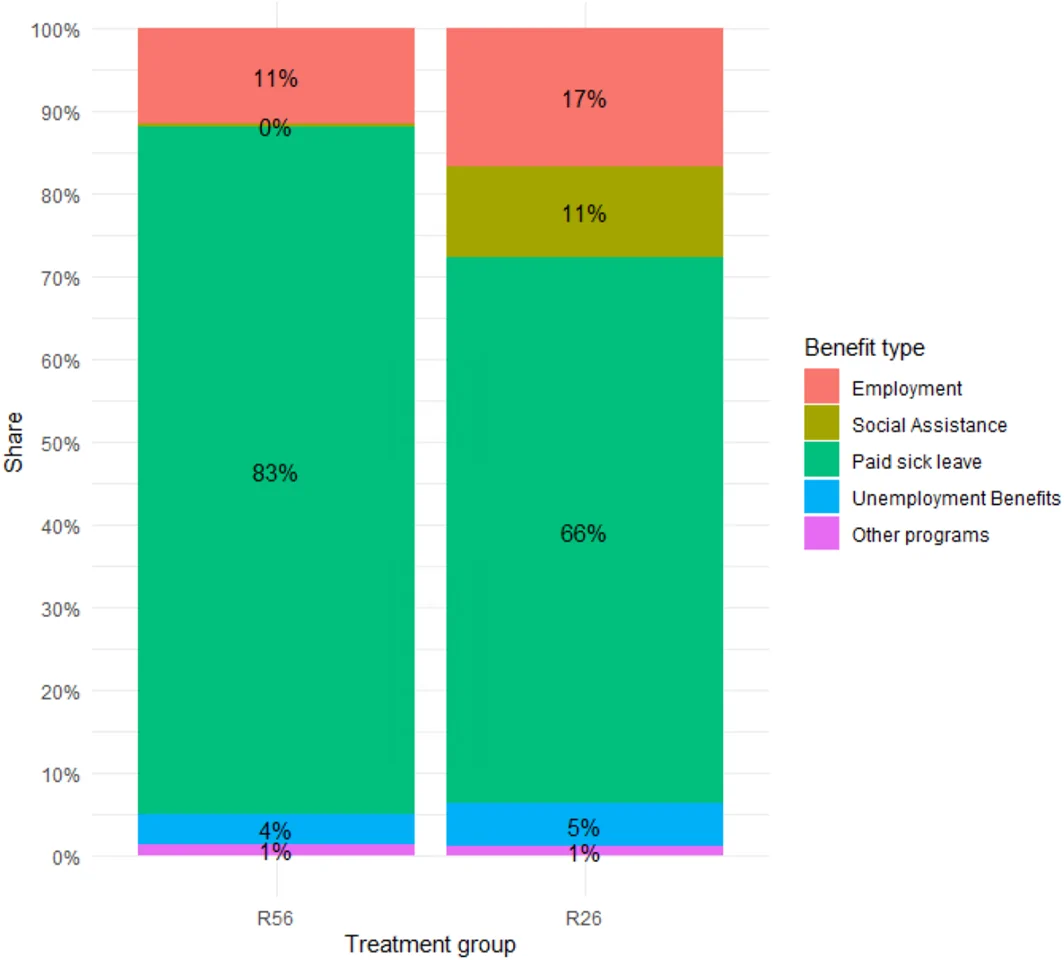
Benefit Types 30 Weeks After Enrollment, Before and After the Reform. This figure shows the distribution of benefit types 30 weeks after enrollment for individuals enrolling before (R56) and after (R26) the reform.

**FIGURE A6:**
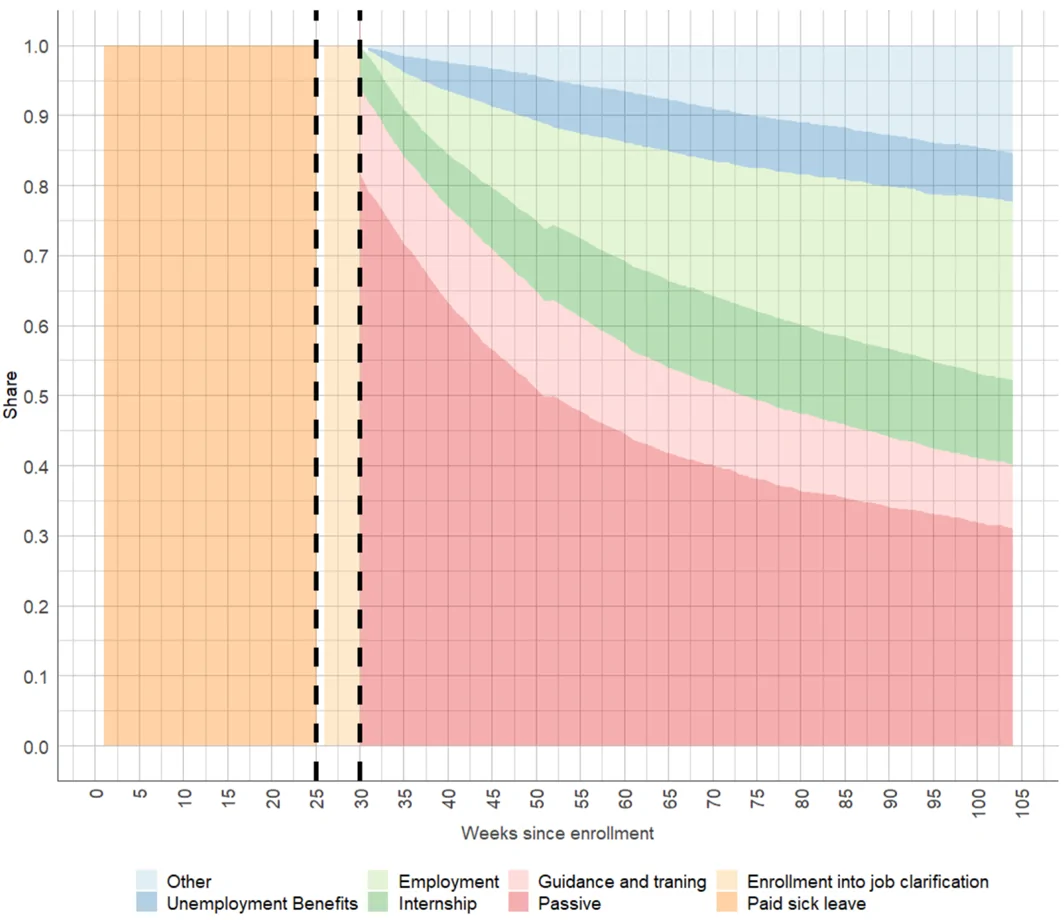
Activation and Employment Transitions Within the Job Clarification Program. The figure shows the distribution of benefit types over time for individuals enrolled in the job clarification program. It illustrates how participants gradually transition from passive support toward employment‐related activation measures and, eventually, employment. The figure includes only employment‐related activities.

**FIGURE A7:**
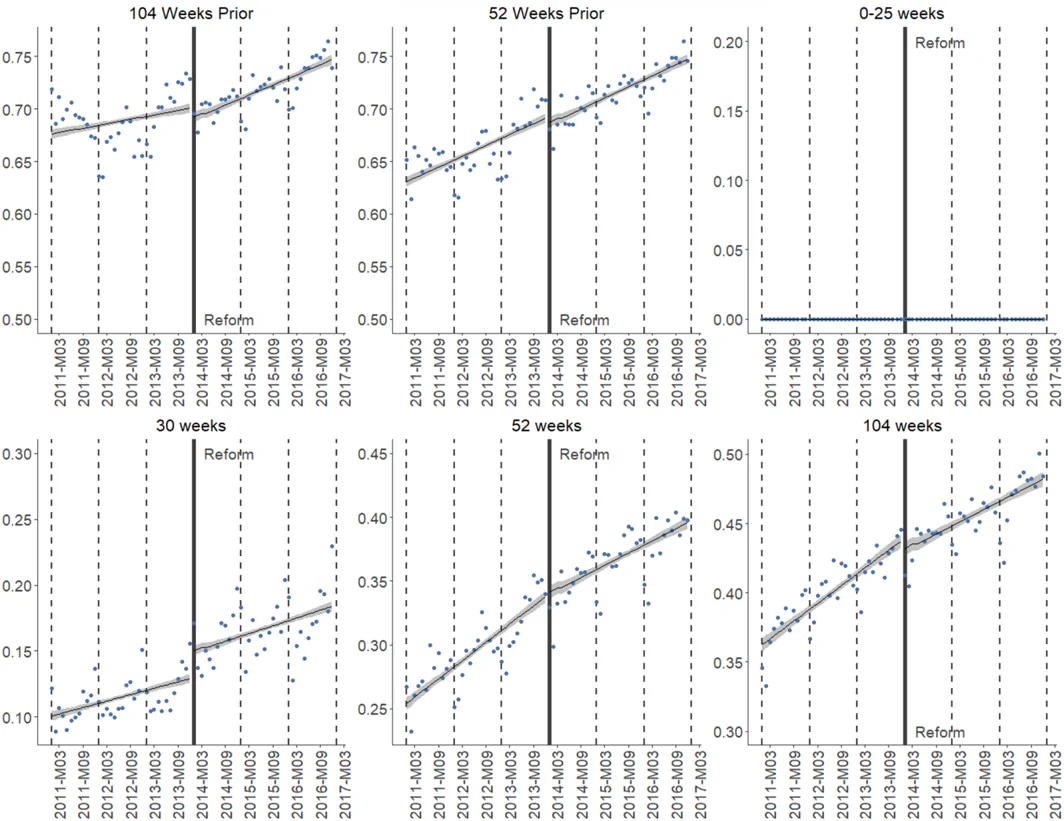
Probability of Employment at Different Time Periods Before and After Enrollment. This figure shows the probability of employment for individuals affected by the reform, depending on the enrollment month. Each panel represents a time period relative to enrollment: 104 weeks before, 52 weeks before, 0–25 weeks after, 30 weeks after, 52 weeks after, and 104 weeks after enrollment. The figure shows no difference in employment probability at 104 or 52 weeks before enrollment across the reform, indicating no selection effects. There is a small short‐term increase in employment probability at 30 weeks after enrollment around the cut‐off. By 52 and 104 weeks, the employment rates are the same across the reform cutoff.

**FIGURE A8:**
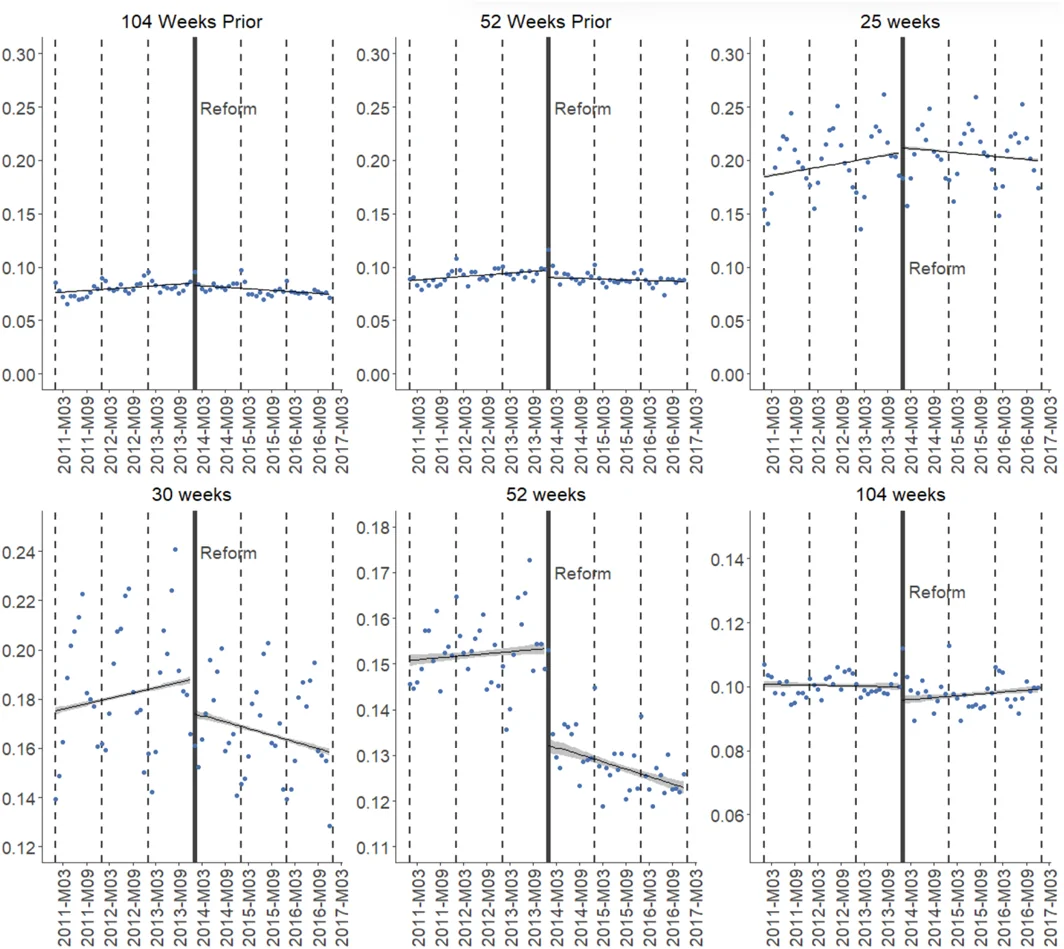
Probability of Being on Paid Sick Leave at Different Time Periods Before and After Enrollment, Full Population. This figure shows the probability of being on paid sick leave for individuals in the full population depending on enrollment month. Each panel represents a time period relative to enrollment: 104 weeks before, 52 weeks before, 25 weeks after, 30 weeks after, 52 weeks after, and 104 weeks after enrollment.

**FIGURE A9:**
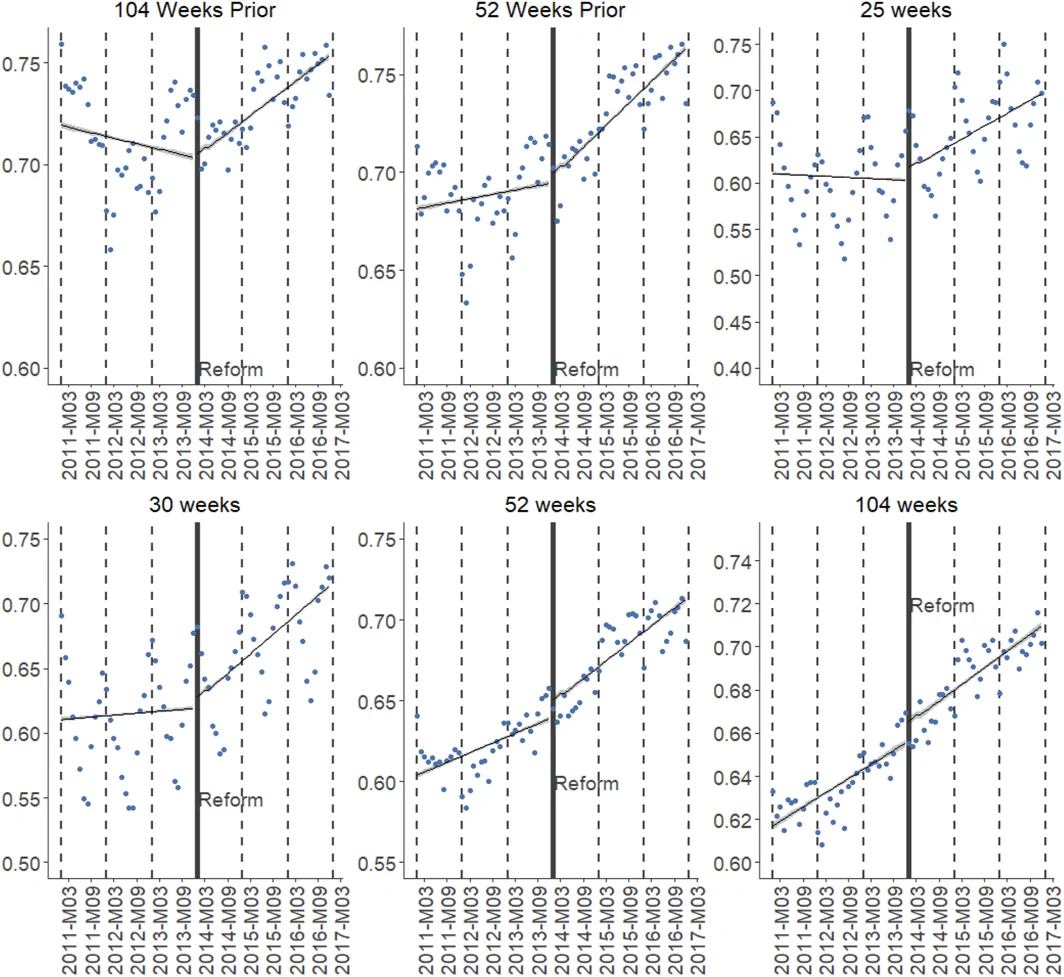
Probability of Employment at Different Time Periods Before and After Enrollment, Full Population. This figure shows the probability of employment for individuals in the full population, depending on the enrollment month. Each panel represents a time period relative to enrollment: 104 weeks before, 52 weeks before, 25 weeks after, 30 weeks after, 52 weeks after, and 104 weeks after enrollment. The figure shows no difference in employment probability at 104 or 52 weeks before enrollment across the reform, indicating no selection effects. There is a small short‐term increase in employment probability at 30 weeks after enrollment around the cut‐off. By 52 and 104 weeks, the employment rates are the same across the reform cut‐.off.

## Data Availability

The data that support the findings of this study are available on request from the corresponding author. The data are not publicly available due to privacy or ethical restrictions.

## Appendix A

### A.1 Validating the Identification Strategy

If individuals strategically manipulate their entry date into paid sick leave to avoid the job clarification program, we would expect to see systematic changes in the distribution of key variables around the time of the reform. Figure A1 presents the mean values of the explanatory variables for individuals enrolling in paid sick leave at different weeks over the years. This is shown for the full sample; however, a similar graph for individuals with a duration of more than 25 weeks is provided in the next subsection.

The vertical line in the figure marks the reform, which separates the two groups. To the left of the line is the “R56” sample, where individuals are reassessed at 56 weeks. To the right of the line is the “R26” sample, where individuals are reassessed at 26 weeks. If individuals were adjusting their enrollment timing to avoid reassessment at 26 weeks, we would expect to see discontinuities in observable characteristics around the reform date.

The figure, however, does not indicate any significant changes in the distribution of explanatory variables across the two groups. The average age of enrollees remains stable at around 40 years. Similarly, the gross income one year prior to enrollment is approximately 300,000 DKK (40,000 EUR), and the fraction of individuals without formal education remains at about 35% throughout the period. While the proportion of males and natives fluctuates slightly over time, there is no systematic pattern associated with the reform. These findings suggest that individuals do not appear to manipulate their entry into the program in response to the change in reassessment timing.

Figure A2 compares the inflow into paid sick leave with and without seasonal adjustment. The top panel shows the raw inflow data, while the bottom panel presents the seasonally adjusted inflow. The unadjusted data in the top panel reveals clear seasonal patterns, with recurring spikes and dips at specific times of the year. There is a noticeable drop in inflow at the end of December during Christmas and New Year, followed by a spike in January. The inflow then gradually declines and reaches its lowest point during the summer holidays. These seasonal patterns likely reflect that individuals rarely report sick during holiday periods, such as Christmas and the summer break.

In contrast, the bottom panel shows the seasonally adjusted inflow, which smooths out these seasonal variations. By removing the seasonal effects, the underlying trend in the data becomes clearer. There is no systematic change in the seasonally adjusted series before and after the reform, suggesting that the timing of inflows into the paid sick leave program is stable and not heavily influenced by manipulation or strategic timing by individuals. Note: For the econometric analysis using the RDD approach, the inclusion of a full calendar year on each side of the cut‐off ensures that any seasonal effects are smoothed out by the linear trends. Therefore, seasonality does not pose a problem for the estimation, as it is mechanical and recurs every year.

### A.2 Balance Test for the Restricted Sample

This subsection presents a figure similar to Figure A1, but specifically for individuals with paid sick leave durations exceeding 25 weeks. The purpose is to validate that key explanatory variables remain balanced around the reform cut‐off within this restricted sample. As shown in Figure A3, the restricted sample also exhibits balance across the reform period, confirming that the identification strategy remains valid for this subset of individuals.

To formally test whether baseline characteristics are balanced around the cutoff, I estimate placebo RD specifications with each covariate as the outcome. Table A1 reports the estimated discontinuities, robust 95% confidence intervals, and *p*‐values. The results show no systematic imbalances, with the exception of a marginally significant difference in age, which likely reflects chance given the number of tests conducted, while the other covariates are statistically indistinguishable across the cutoff.

**TABLE A1:** Balance tests around the cutoff.

Variable	Coef.	95% CI lower	95% CI upper	*p*‐value
Age	−0.799	−1.570	−0.032	0.041
Male	−0.016	−0.053	0.022	0.421
Native	−0.005	−0.030	0.019	0.658
Education	0.032	−0.006	0.069	0.102
Income	−9110	−20490	2269	0.117

The final aspect to examine is whether systematic manipulation occurs around the cut‐off. Figure A4 shows the density of the running variable, measured in months from the reform cut‐off on January 1, 2014. There is a visible difference in density around the cut‐off, particularly within the ± 12‐month bandwidth. The linear fit within this range shows systematically fewer entries into paid sick leave during the first half of the year compared to the second half, resulting in a slight drop in density right after the cut‐off. However, the slopes on both sides of the cut‐off are identical, indicating that this pattern reflects a purely mechanical seasonal effect rather than any manipulation. When expanding the bandwidth to ± 24 months, the slope becomes flatter, and with a bandwidth of ± 36 months, the seasonal variation is fully smoothed out, leaving no visible difference in density on either side of the cut‐off.

To formally test for manipulation at the cut‐off, I estimate discontinuities in the log‐density of the running variable using different bandwidths. The results, reported in Table A2, show no statistically significant discontinuities at the reform threshold. The estimate using the narrowest bandwidth of ± 12 months is close to being statistically significant, which reflects the mechanical seasonal drop in density around January rather than any manipulation of the running variable. For wider bandwidths, the estimates flatten and become entirely insignificant, confirming that the observed variation is due to seasonality.

**TABLE A2:** McCrary test for discontinuity in log‐density at the cut‐off.

Bandwidth (months)	Estimate (Δ)	SE(Δ)	*t*‐stat	*p*‐value
±12	−0.216	0.133	−1.62	0.105
±24	−0.087	0.101	−0.86	0.389
±36	−0.046	0.091	−0.50	0.616

*Note:* The table reports McCrary‐type discontinuity tests in the **log‐density** of the running variable at the reform cut‐off. None of the estimated discontinuities are statistically significant. The estimate for the ±12‐month bandwidth is close to significance, which is explained by a seasonal dip in density around January rather than manipulation.

### A.3 Understanding the Complier Group

To shed light on the complier group, that is those individuals whose probability shifts such that they no longer remain on paid sick leave, Figure A5 shows the distribution of benefit types 30 weeks after enrollment for individuals before (R56) and after (R26) the reform. The figure demonstrates that the reform‐induced decline in paid sick leave of roughly 17% points is mirrored by an increase in participation in the job clarification program (classified as social assistance) from 0 to 11%, and by an increase in employment from 11 to 17%. Together, these changes fully account for the 17% point shift, illustrating how the complier group is reallocated between activation and employment.

To better understand what happens to individuals who do not return directly to employment but instead enter the job clarification program—i.e., the 11% shown in Figure A5—Figure A6 presents their subsequent trajectories and the types of employment‐related activities they participate in. The figure includes only employment‐related activation measures—such as guidance, training, or internships—and does not capture potential social or health‐related activities (e.g., psychological treatment), which are not available in the data. Hence, the figure represents a lower‐bound estimate of activation intensity.

From week 1 up to week 26, individuals are on paid sick leave, with week 26 marking the reassessment point introduced by the reform. Between weeks 26 and 30, individuals transition from paid sick leave to the job clarification program, as illustrated by the vertical dashed lines in the figure.

The figure shows that 30 weeks after enrollment into paid sick leave—corresponding to the beginning of the job clarification spell—82% of participants in the job clarification program are passive, receiving no employment‐related activation. Around 12% participate in guidance and training activities, while 6% are in internships. After one year, the share of passive participants declines to about 50%, whereas 14% receive guidance and training, 11% are in internships, and 14% have transitioned to employment. The remaining participants are either on unemployment benefits (5%) or classified as “other” (5%), where the latter category includes programs such as old‐age pension and disability insurance.

After two years, the share of passive participants further declines to 31%. About 25% are in employment, 9% receive guidance and training, 12% are in internships, 7% are on unemployment benefits, and 15% fall into the “other” category.

After documenting how individuals in the job clarification program gradually transition toward employment‐related activities, the next step is to examine whether these transitions translate into measurable changes in employment for the broader group affected by the reform.

### A.4 Reduced‐Form Effect on Employment

Figure A7 shows the probability of employment over time for individuals affected by the reform, that is the reduced‐form effect on employment. Again, the panels represent different time periods relative to enrollment.

At 104 weeks prior to enrollment, the probability of employment is around 70%, with a steady increasing trend. Importantly, there is no noticeable difference in employment probability 104 weeks before enrollment when comparing groups across the reform. A similar pattern is observed in the figure showing 52 weeks prior to enrollment, indicating that no systematic selection effects are present.

By definition, because individuals in the sample are enrolled in paid sick leave during the first 25 weeks after enrollment, the upper‐right panel shows that the probability of employment is zero in this period.

The lower panels show a shift in the probability of employment for individuals enrolling just after the reform compared to those enrolling before. This represents the reduced‐form effect for the entire population, not only for the complier group—the roughly 17% points who no longer remain on paid sick leave due to the reform. Specifically, 30 weeks after enrollment, the probability of employment is a few percentage points higher around the reform cut‐off. Interestingly, this effect is measured right at the onset of the job clarification program (see Figure A6). At this point, activation activities have barely started, so the uptick in employment cannot be attributed to program participation. Instead, it likely reflects a threat effect: individuals choosing to return to work rather than entering the new program. By 52 weeks, the employment rates are the same. This confirms the findings from the previous section—many of the individuals enrolled in the job clarification program would have naturally returned to employment in the following weeks.

Interestingly, the program also does not show any long‐term effects. At 104 weeks, the employment rates around the cut‐off are almost identical. That is, there appear to be no lasting effects of the reform. This further supports the conclusion that the employment increase is not driven by lasting improvements in labor market attachment but rather by individuals reacting to the activation requirement.

Next, I proceed to formally estimate the effect of the reform.

Table A3 presents the reduced form estimates from the regression discontinuity design. The coefficients vary moderately across bandwidths and polynomial orders but remain highly consistent across kernel choices. As in the instrumental variable results, the variation across specifications primarily reflects seasonal fluctuations in employment and paid sick leave around the cut‐off, as illustrated in Figure A7. When narrow bandwidths are used, the estimates become sensitive to whether the sample includes complete calendar years on both sides of the reform. Similarly, higher‐order polynomials tend to overfit these seasonal patterns, resulting in smaller or even negative estimates. Using a linear specification and a bandwidth of approximately 3 years on each side of the reform effectively smooths out such seasonality.

**TABLE A3:** Reduced form estimates under different specifications.

	12 months	24 months	36 months
Bandwidth			
Coefficient	−0.009	0.010	0.018
*t*‐value	−1.76	2.99	6.38

	**Linear**	**Quadratic**	**Cubic**
Polynomial order			
Coefficient	0.018	0.005	−0.012
*t‐*value	6.38	1.15	−2.06

	**Triangular**	**Uniform**	**Epanechnikov**
Kernel function			
Coefficient	0.018	0.019	0.020
*t*‐value	6.38	7.36	7.38

*Note:* The table reports reduced form estimates of the effect of the 2014 reform on the probability of employment 30 weeks after enrollment for individuals with sick‐leave spells longer than 25 weeks. All specifications control for age, gender, being native‐born, and education. Based on 143,819 observations.

In terms of magnitude, the preferred specification with a 36‐month bandwidth and a linear functional form indicates that the 2014 reform increased the probability of employment by about 1.8% points 30 weeks after enrollment. This estimate captures the overall or reduced‐form effect of the reform on employment, reflecting both the direct mechanical impact of the reassessment rule and any behavioral adjustments in response to the policy change.

### A.5 Full Sample Analysis

In the main text, I restricted the analysis to spells lasting longer than 25 weeks, that is, individuals at risk. In this appendix section, I instead consider the full population. The motivation is that if individuals responded to the reform already before the reassessment at week 26, this could generate selection bias in the main analysis. Figure A8 displays the probability of being on paid sick leave at different points in time relative to enrollment—104 weeks before, 52 weeks before, 25 weeks after, 30 weeks after, 52 weeks after, and 104 weeks after—depending on the calendar time of entry.

The two panels in the top left show the probability of being on sick leave 104 and 52 weeks prior to the current spell. The probabilities are similar across the reform, around seven to nine percent, suggesting that the two samples are balanced with respect to prior sick leave histories.

The top‐right panel displays the probability of being on sick leave at week 25 for the full population. If individuals had responded to the reform before the reassessment at week 26, we would expect a lower probability of being on sick leave 25 weeks after enrollment. Instead, the probability is virtually identical before and after the reform, indicating no evidence of anticipatory selection. This lack of selection is likely due to the fact that individuals only learn at the reassessment at week 26 whether their paid sick leave will be extended.

Finally, the figure shows that the probability of being on sick leave at weeks 30 and 52 is lower after the reform. These effects are less pronounced than in the main analysis, since the sample here is not restricted to individuals with spells lasting longer than 25 weeks.

Figure A9 shows the probability of employment at different points in time relative to enrollment for the full population. While the figure suggests an increase in employment around 30 and 52 weeks after enrollment, the effects are difficult to detect visually. This is because only about 15% of all individuals remain on sick leave beyond 25 weeks and are thus actually subject to the new reassessment rule. Moreover, as discussed in the main text, only 17% points of this group are directly affected by the reform.

## References

[hec70126-bib-0001] Calonico, S. , M. D. Cattaneo , and R. Titiunik . 2014. “Robust Nonparametric Confidence Intervals for Regression‐Discontinuity Designs.” Econometrica 82, no. 6: 2295–2326. 10.3982/ecta11757.

[hec70126-bib-0002] Card, D. , J. Kluve , and A. Weber . 2010. “Active Labour Market Policy Evaluations: A Meta‐Analysis.” Economic Journal 120, no. 548: F452–F477. 10.1111/j.1468-0297.2010.02387.x.

[hec70126-bib-0003] Dale‐Olsen, H. 2014. “Sickness Absence, Sick Leave Pay, and Pay Schemes.” Labour 28, no. 1: 40–63. 10.1111/labr.12022.

[hec70126-bib-0004] D’Amuri, F. 2017. “Monitoring and Disincentives in Containing Paid Sick Leave.” Labour Economics 49: 74–83. 10.1016/j.labeco.2017.09.004.

[hec70126-bib-0005] De, P. , V. Scoppa , and V. Pupo . 2014. “Absenteeism in the Italian Public Sector: The Effects of Changes in Sick Leave Policy.” Journal of Labor Economics 32, no. 2: 337–360. 10.1086/674986.

[hec70126-bib-0006] Department for Work & Pensions . 2008. The Incapacity Benefit Reform Project (Amendment) Regulations 2008: Explanatory Memorandum.

[hec70126-bib-0007] Fevang, E. , S. Markussen , and K. Røed . 2014. “The Sick Pay Trap.” Journal of Labor Economics 32, no. 2: 305–336. 10.1086/673400.

[hec70126-bib-0008] Graversen, B. K. , and J. C. van Ours . 2008. “How to Help Unemployed Find Jobs Quickly: Experimental Evidence From a Mandatory Activation Program.” Journal of Public Economics 92, no. 10: 2020–2035. 10.1016/j.jpubeco.2008.04.013.

[hec70126-bib-0009] Henrekson, M. , and M. Persson . 2004. “The Effects on Sick Leave of Changes in the Sickness Insurance System.” Journal of Labor Economics 22, no. 1: 87–113. 10.1086/380404.

[hec70126-bib-0010] Imbens, G. W. , and L. Thomas . 2008. “Regression Discontinuity Designs: A Guide to Practice.” Journal of Econometrics 142, no. 2: 615–635. 10.1016/j.jeconom.2007.05.001.

[hec70126-bib-0011] Johansson, P. , and M. Palme . 2005. “Moral Hazard and Sickness Insurance.” Journal of Public Economics 89, no. 9: 1879–1890. 10.1016/j.jpubeco.2004.11.007.

[hec70126-bib-0012] Lalive, R. , J. van Ours , and J. Zweimüller . 2006. “How Changes in Financial Incentives Affect the Duration of Unemployment.” Review of Economic Studies 73, no. 4: 1009–1038. 10.1111/j.1467-937x.2006.00406.x.

[hec70126-bib-0013] Markussen, S. , K. Røed , O. J. Røgeberg , and S. Gaure . 2011. “The Anatomy of Absenteeism.” Journal of Health Economics 30, no. 2: 277–292. 10.1016/j.jhealeco.2010.12.003. 21247647

[hec70126-bib-0014] Nekoei, A. , and A. Weber . 2017. “Does Extending Unemployment Benefits Improve Job Quality?” American Economic Review 107, no. 2: 527–561. 10.1257/aer.20150528.

[hec70126-bib-0015] Pettersson‐Lidbom, P. , and P. S. Thoursie . 2013. “Temporary Disability Insurance and Labor Supply: Evidence From a Natural Experiment.” Scandinavian Journal of Economics 115, no. 2: 485–507. 10.1111/j.1467-9442.2012.01746.x.

[hec70126-bib-0016] Pichler, S. 2015. “Sickness Absence, Moral Hazard, and the Business Cycle.” Health Economics 24, no. 6: 692–710. 10.1002/hec.3054. 24737552

[hec70126-bib-0017] Puhani, P. A. , and K. Sonderhof . 2010. “The Effects of a Sick Pay Reform on Absence and on Health‐Related Outcomes.” Journal of Health Economics 29, no. 2: 285–302. 10.1016/j.jhealeco.2010.01.003. 20153537

[hec70126-bib-0018] Regeringen . 2007. Införande Av En Rehabiliteringskedja.

[hec70126-bib-0019] Rehwald, K. , M. Rosholm , and B. Rouland . 2018. “Labour Market Effects of Activating Sick‐Listed Workers.” Labour Economics 53: 15–32. 10.1016/j.labeco.2018.04.003.

[hec70126-bib-0020] Statistics Denmark . 2022. Statistisk Tiårsoversigt 2022. DST.

[hec70126-bib-0021] Stearns, J. , and C. White . 2018. “Can Paid Sick Leave Mandates Reduce Leave‐Taking?” Labour Economics 51: 227–246. 10.1016/j.labeco.2018.01.002.

[hec70126-bib-0022] Ziebarth, N. R. 2013. “Long‐Term Absenteeism and Moral Hazard—Evidence From a Natural Experiment.” Labour Economics 24: 277–292. 10.1016/j.labeco.2013.09.004.

[hec70126-bib-0023] Ziebarth, N. R. , and M. Karlsson . 2010. “A Natural Experiment on Sick Pay Cuts, Sickness Absence, and Labor Costs.” Journal of Public Economics 94, no. 11: 1108–1122. 10.1016/j.jpubeco.2010.09.001.

[hec70126-bib-0024] Ziebarth, N. R. , and M. Karlsson . 2014. “The Effects of Expanding the Generosity of the Statutory Sickness Insurance System.” Journal of Applied Econometrics 29, no. 2: 208–230. 10.1002/jae.2317.

